# The generalized Wiener–Hopf equations for wave motion in angular regions: electromagnetic application

**DOI:** 10.1098/rspa.2021.0040

**Published:** 2021-08

**Authors:** V. G. Daniele, G. Lombardi

**Affiliations:** Department of Electronics and Communications, Politecnico di Torino, 10129 Torino, Italy

**Keywords:** wave motion, wedge, Wiener–Hopf method, integral equations, spectral domain, electromagnetics

## Abstract

In this work, we introduce a general method to deduce spectral functional equations and, thus, the generalized Wiener–Hopf equations (GWHEs) for wave motion in angular regions filled by arbitrary linear homogeneous media and illuminated by sources localized at infinity with application to electromagnetics. The functional equations are obtained by solving vector differential equations of first order that model the problem. The application of the boundary conditions to the functional equations yields GWHEs for practical problems. This paper shows the general theory and the validity of GWHEs in the context of electromagnetic applications with respect to the current literature. Extension to scattering problems by wedges in arbitrarily linear media in different physics will be presented in future works.

## 1. Introduction

The extension of the Wiener–Hopf (WH) technique in angular regions [1–5] demonstrated its efficacy for solving electromagnetic wave-scattering problems in the presence of geometries containing angular regions and/or stratified planar regions; see for instance [6–10] and references therein.

This technique consists of three steps: (i) the deduction of functional equations in the spectral domain of subregions that constitute the whole geometry of the problem, (ii) the imposition of boundary conditions to get the generalized Wiener–Hopf equations (GWHEs), and (iii) the solution of the system of the WH equations using exact or semi-analytical/approximate techniques of factorization, such as the Fredholm factorization technique [5–12].

This paper is focused on the first and second steps of the procedure and shows a new general methodology. In particular, we deduce spectral functional equations and GWHEs for angular regions filled by arbitrary linear homogeneous media in a general framework, following the procedure first proposed in [3] with applications to electromagnetics.

The introduction of the GWHEs in angular regions was inspired by Vekua in [13]. This book introduces the Hilbert generalized equations and shows that, with slight modifications, these equations can be solved using the same procedures developed for the solution of the functional equation for classical Hilbert problems. We note that these equations are more general than those defined in the WH method.

The GWHEs differ from the classical Wiener–Hopf equations (CWHEs) in terms of the definitions of the unknowns in the spectral domain. While CWHEs introduce plus and minus functions that are always defined in the same complex plane, the GWHEs present plus and minus functions that are defined in different complex planes but that are related. However, in several important practical cases, suitable mappings allow the plus and minus functions of GWHEs to be redefined in the same complex plane: for instance, in angular subregions see the mapping reported in [1–6]. With this transformation we ensure the remarkable property that GWHEs reduce to CWHEs.

When the problem can be formulated in terms of Helmholtz equations, the GWHEs are related to the difference equation of the Sommerfeld–Malyuzhinets (SM) method; see for instance, in wedge problem [10] and references therein. In particular, the mapping η=−kcos⁡w relates the spectral variables *η* and *w*, respectively defined in the WH equations using the Laplace transform and in the difference equations using the SM method. Passing from the *η* plane to the *w* plane (and vice versa) is an expedient that allows us to exploit solution properties of the same problem using two methods (the WH factorization technique and SM difference equations). Hence, the analysis of problems with the SM and WH methods have a useful synergy. This means that the study of scattering problems in the presence of angular regions with different methods is fundamental. In particular, important improvements on the SM method are reported in the books by Babich *et al.* [14], Bernard [15], Budaev [16], Lyalinov & Zhu [17] and references therein.

The introduction of the GWHEs in scattering problems by angular regions presents some aspects in common with the study of right-bounded regions; see [5,10] and references therein. In particular, several works on right-angled structures have been studied in terms of the Riemann–Hilbert (RH) formulations [18–20], and the relationship between RH and WH methods may be examined in depth. However, WH and/or RH formulations of angular regions have rarely been considered in the literature and fully interpreted. For the WH method, the last equation of example 5.15 in [21] is a GWHE. In particular, Noble [21] suggested the mapping η=−kcos⁡w as a natural substitution to obtain the solution.

We also observe that Gautesen in numerous papers (e.g. [22–25]) proposed the solution of the fundamental scattering problem from an elastic wedge, where the functional angular equations are substantially GWHEs although they are not defined in this way. This author provides efficient semi-analytical solutions of the spectral equations using the Cauchy decomposition formula in the spectral plane. His method can be considered an efficient technique to approximately solve GWHEs.

GWHEs were also introduced in [26,27] to solve the electromagnetic scattering problem of a perfectly electrical conducting (PEC) wedge as well as of an impedance wedge. These authors are aware that their equations might be dealt with using the factorization technique; however, they proposed a solution based on the SM method and difference equations.

A last set of works concerning the introduction of GWHEs in wedge problems is [28,29]. The novelty of these works resides in the application of a mapping that provides a factorization method to solve difference equations in the SM method for acoustic impenetrable wedge scalar scattering problems. We recall also that the factorization method to solve difference equations was, for example, proposed in [30]. We note that the mapping used in [28] resembles the one introduced in [1–6] but the motivation of its introduction is different. In particular, in [1–6], the mapping is introduced to systematically reduce GWHEs of general angular-shaped region wave problems defined in the Laplace domain to the usual classical WH equations [21].

As per rectangular regions, the WH equations of scattering problems in angular regions can be obtained using two strategies. The first method consists of formulating the problem in terms of integral equations in the natural domain using suitable Green’s functions [31]. Since the formulation contains integral representations with convolutional kernels the application of the Fourier or Laplace transforms yields the WH equations in the spectral domain. The second method to obtain the WH equations in the spectral domain is proposed by Jones [32] and Noble [21]. It is based on the application of the Fourier or Laplace transforms directly to the partial differential equation formulation of the problem, avoiding the necessity to study the Green’s function representations in the natural domain. The Jones procedure is convenient, flexible and applicable to arbitrary media and physics where the evaluation of Green’s function can constitute a cumbersome difficult problem. While the deduction of the functional equations in [22–29] is based on the first method also using the second Green’s identity, we propose in this paper to use the Jones method. We note that, in order to apply Jones’s approach to get the GWHEs in the presence of angular region problems, it is important to introduce partial differential equation formulations using oblique Cartesian coordinates, as in [1–5].

We have developed different strategies to apply Jones’s method. In this paper, we use a novel general first-order differential vector formulation for transverse components of the fields as in [3,4] and as first proposed in [33,34] as a method for solving for rectangular problems. The method differs from the one reported in [1,2,5], where the second-order differential formulation (wave equation) is applied. We claim superiority of the new procedure (based on the first-order formulation) to obtain spectral functional equations in angular regions, since it is capable of modelling arbitrary linear media in systematic steps, as illustrated in the paper. Derivation of the explicit equations requires implementation of the procedure reported in the paper, which is illustrated explicitly for isotropic media and is extendable to more complex media (e.g. appendix A). While the first-order procedure provides a method to obtain the functional equations for general arbitrary linear media filling the angular region, we note that the second-order formulation [2,5] is impractical in non-isotropic media since no systematic procedural steps are available. Moreover, the first-order differential formulation can also be extended to wave motion problems in different physics.

In this paper, plane-wave sources and/or sources localized at infinity are considered in a time-harmonic electromagnetic field with a time dependence specified by ${\text{e}}^{j\omega t}$ (electrical engineering notation), which is suppressed. The paper is organized into six sections, two appendices and a glossary. The deduction of the GWHEs for scattering problems by wedges in an arbitrary linear homogeneous medium is based on applying the boundary conditions to relevant spectral functional equations of angular regions. The main aim of this paper is to obtain these functional equations by introducing a conceptually simple technique starting from a first-order differential vector formulation in terms of transverse components of fields (transverse equations). In order to develop this technique, a preliminary study based on an abstract formulation of Maxwell’s equations in an indefinite homogeneous medium is necessary, as reported in §2. We recall that this methodology is also useful to study propagation in stratified media.

Using oblique Cartesian coordinates and taking into account the results of §2, §3 describes the novel application of the method to angular regions with oblique Cartesian coordinates, yielding the oblique transverse equations. The solution of these oblique transverse equations (§3), projected on the reciprocal eigenvectors of an algebraic matrix defined in §2, provides the functional equations of an arbitrary angular region, reported in §4. It is remarkable that we get functional equations independently from the materials and the sources that can be present outside the considered angular region. The properties and validations of functional equations and how to get the GWHEs by imposing the boundary conditions on the two faces of the angular region are finally reported in §5 for isotropic media, with conclusions in §6. Appendix A reports fundamental explicit matrices to apply the methodology to anisotropic media, while appendix B justifies the dyadic Green’s function formula of §4. The glossary reports the main abbreviations, notations and symbols and is useful for the readability of the text.

## 2. First-order differential transverse equations for indefinite rectangular regions filled by arbitrary linear homogeneous media

The evaluation of the physical fields in a linear medium can be generally described by a system of partial differential equations of first order. In the absence of sources localized at finite or in the presence of plane-wave sources, the system assumes the homogeneous abstract form

2.1 $$\Gamma_{\nabla }\cdot \psi =\theta ,$$

where $\Gamma_{\nabla }$ is a matrix differential operator that contains partial derivatives of first order, *ψ* is a vector that defines the field to be evaluated and *θ* is an additional field that is related to the field *ψ* through constitutive relations depending on the parameters that define the physical characteristics of the medium where the field is considered. *ψ* and *θ* are vectors that have the same dimensions and the constitutive relations are defined by

2.2 θ=W⋅ψ,

where the matrix *W* depends on the medium that is considered.

In electromagnetism, the fields **E** and **H** in an arbitrary homogeneous linear medium are governed by Maxwell’s equations and present the following constitutive relations:

2.3 $$\begin{array}{rl} & \text{D}=\varepsilon \cdot \text{E}+\xi \cdot \text{H}\\ \;\text{and}\; & \text{B}=\zeta \cdot \text{E}+\mu \cdot \text{H}. \end{array}\}$$

Thus, in electromagnetic applications, (2.1) and (2.2) are defined by

2.4 $$\psi =\left|\begin{array}{c} \text{E}\\ \text{H} \end{array}\right|,\;\theta =j\omega \left|\begin{array}{c} \text{D}\\ -\text{B} \end{array}\right|,\;\Gamma_{\nabla }=\left|\begin{array}{cc} 0 & \nabla \times 1\\ \nabla \times 1 & 0 \end{array}\right|\;\text{and}\;W=j\omega \left|\begin{array}{cc} \varepsilon & \xi \\ -\zeta & -\mu \end{array}\right|,$$

where **1** is the unit dyadic in the Euclidean space. An extended and detailed treatise about this abstract formulation is reported in [35], but this is not easily accessible and is not well known in the scientific community; for this reason, here we give a short introduction and then our application.

To complete the formulation of the field problem via (2.1)–(2.4), we also need to impose the geometrical domain of the problem, its boundary conditions and the radiation condition.

In our method, first, we derive spectral functional equations avoiding the application of boundary conditions for a particular domain and, then, in practical problems, we impose the boundary conditions coupling different regions and yielding the GWHEs of the problem.

For this reason, in the following sections, the boundary conditions will appear only at §5 where a practical classical problem will be examined as an example of the implementation procedure: the Malyuzhinets problem.

The application of the abstract formulation to the electromagnetic study of the stratified medium along a direction (say *y*) is fundamental to introducing several important concepts in wave propagation (e.g. [36,37]). In particular, the introduction of the transverse equations can be used for the analysis of indefinite regions and in §3 for the development of the theory for angular regions. The transverse equations of a field are equations that involve only the components of the field *ψ*, say *ψ*_*t*_, that remain continuous along the stratification according to the boundary conditions on the interfaces. In [35], the abstract deduction of the transverse equations is obtained starting from the abstract equations (2.1) and (2.2).

In the following, we assume y=const. in Cartesian coordinates as the interface among media of rectangular shape (layers). To obtain the boundary conditions, the method resorts to a suitable application of the divergence theorem on equation (2.1) (e.g. [33]). In electromagnetism, the transverse field for a stratification along the *y*-direction is

2.5 $$\psi_{t}=|{\text{E}}_{t}\;{\text{H}}_{t}{|}^{\prime }=|E_{z}\;E_{x}\;H_{z}\;H_{x}{|}^{\prime },$$

where ′ stands for transpose and ${\text{E}}_{t}=\hat{z}E_{z}+\hat{x}E_{x},{\text{H}}_{t}=\hat{z}H_{z}+\hat{x}H_{x}$ satisfy the boundary condition of continuity on the interfaces of the stratification.

Following [35], we deduce the electromagnetic transverse equations with respect to *y*, starting from (2.1)–(2.4) for a general bianisotropic medium with constitutive parameters *W* where ε,ξ,ζ,μ are tensors. For practical evaluation, we assume Cartesian coordinates with the ordering (z,x,y). We start from the decomposition of the differential operator

2.6 $$\nabla =\nabla_{t}+\hat{y}\frac{\partial }{\partial y},\;\nabla_{t}=\hat{z}\frac{\partial }{\partial z}+\hat{x}\frac{\partial }{\partial x},$$

which yields

2.7 $$\Gamma_{\nabla }=\Gamma_{t}+\Gamma_{y}\frac{\partial }{\partial y}$$

with

2.8 $$\Gamma_{t}=\left|\begin{array}{cc} 0 & \nabla_{t}\times 1\\ \nabla_{t}\times 1 & 0 \end{array}\right|,\;\Gamma_{y}=\left|\begin{array}{cc} 0 & \hat{y}\times 1\\ \hat{y}\times 1 & 0 \end{array}\right|,\;1=\hat{z}\hat{z}+\hat{x}\hat{x}+\hat{y}\hat{y}.$$

We observe that the following dyadic relations hold:

2.9 $$I_{t}\cdot \Gamma_{t}=\Gamma_{t}\cdot I_{y},\;I_{t}\cdot \Gamma_{y}=\Gamma_{y}\cdot I_{t}=\Gamma_{y},\;I_{y}\cdot \Gamma_{t}=\Gamma_{t}\cdot I_{t}\;\text{and}\;I_{y}\cdot \Gamma_{y}=\Gamma_{y}\cdot I_{y}=0,$$

where

2.10 $$I_{t}=\left|\begin{array}{cc} 1_{t} & 0\\ 0 & 1_{t} \end{array}\right|,\;I_{y}=\left|\begin{array}{cc} 1_{y} & 0\\ 0 & 1_{y} \end{array}\right|,\;1_{t}=\hat{z}\hat{z}+\hat{x}\hat{x},\;1_{y}=\hat{y}\hat{y}.$$

Taking into account (2.6)–(2.10), the first member of (2.1) becomes

2.11 $$\Gamma_{\nabla }\cdot \psi =\left(\Gamma_{t}+\Gamma_{y}\frac{\partial }{\partial y}\right)\psi =\Gamma_{t}\cdot \psi_{t}+\Gamma_{y}\frac{\partial }{\partial y}\psi_{t}+\Gamma_{t}\cdot \psi_{y},$$

where $\psi_{t}=|E_{t}\;H_{t}{|}^{\prime }=|E_{z}\;E_{x}\;H_{z}\;H_{x}{|}^{\prime }$ and $\psi_{y}=|E_{y}\hat{y}\;H_{y}\hat{y}{|}^{\prime }$ with ${\text{E}}_{t}=\hat{z}E_{z}+\hat{x}E_{x},{\text{H}}_{t}=\hat{z}H_{z}+\hat{x}H_{x}$.

Using the representation

2.12 $$W=W_{tt}+W_{ty}+W_{yt}+W_{yy},$$

where $W_{tt}=I_{t}\cdot W\cdot I_{t}$, $W_{ty}=I_{t}\cdot W\cdot I_{y}$, $W_{yt}=I_{y}\cdot W\cdot I_{t}$, $W_{yy}=I_{y}\cdot W\cdot I_{y}$, we have the following decomposition in transversal and longitudinal components of (2.1):

2.13 $$I_{y}\cdot \Gamma_{t}\cdot \psi_{t}=W_{yt}\cdot \psi_{t}+W_{yy}\cdot \psi_{y}$$

and

2.14 $$I_{t}\cdot \frac{\partial }{\partial y}\Gamma_{y}\cdot \psi_{t}+I_{t}\cdot \Gamma_{t}\cdot \psi_{y}=W_{tt}\cdot \psi_{t}+W_{ty}\cdot \psi_{y}.$$

By substituting the matrix ${\hat{W}}_{y}$ defined by

2.15 $${\hat{W}}_{y}\cdot W_{yy}=W_{yy}\cdot {\hat{W}}_{y}=I_{y}$$

into (2.13), it yields the relation that connects the longitudinal field $\psi_{y}$ in terms of the transversal field $\psi_{t}$

2.16 $$\psi_{y}={\hat{W}}_{y}\cdot (I_{y}\cdot \Gamma_{t}-W_{yt})\cdot \psi_{t},$$

where explicitly

2.17 $${\hat{W}}_{y}=\frac{1}{j\omega (\varepsilon_{y}\mu_{y}-\xi_{y}\zeta_{y})}\left|\begin{array}{cc} \mu_{y}1_{y} & \xi_{y}1_{y}\;\\ -\zeta_{y}1_{y} & -\varepsilon_{y}1_{y} \end{array}\right|.$$

Taking into account that $\Gamma_{y}^{2}=-I_{t}$, the substitution of (2.16) into (2.14) yields the transversal Maxwell equations (2.18)

2.18 −∂∂yψt=M(∂∂z,∂∂x)⋅ψt,

where the matrix operator of dimension 4, M(∂/∂z,∂/∂x), is given by

2.19 $$M\left(\frac{\partial }{\partial z},\frac{\partial }{\partial x}\right)=-\Gamma_{y}\cdot [I_{t}\cdot (\Gamma_{t}-W_{ty})\cdot {\hat{W}}_{y}\cdot (I_{y}\cdot \Gamma_{t}-W_{yt})-W_{tt}].$$

In the case of an isotropic medium, i.e. with

2.20 $$W=j\omega \left|\begin{array}{cc} \varepsilon \text{1} & 0\\ 0 & -\mu \text{1} \end{array}\right|,$$

we obtain

2.21 $$\begin{array}{r} M\left(\frac{\partial }{\partial z},\frac{\partial }{\partial x}\right)=\left(\begin{array}{cccc} 0 & 0 & -\frac{jD_{x}D_{z}}{\varepsilon \omega } & \frac{j({D_{z}}^{2}+\varepsilon \mu \omega^{2})}{\varepsilon \omega }\\ 0 & 0 & -\frac{j({D_{x}}^{2}+\varepsilon \mu \omega^{2})}{\varepsilon \omega } & \frac{jD_{x}D_{z}}{\varepsilon \omega }\\ \frac{jD_{x}D_{z}}{\mu \omega } & -\frac{j({D_{z}}^{2}+\varepsilon \mu \omega^{2})}{\mu \omega } & 0 & 0\\ \frac{j({D_{x}}^{2}+\varepsilon \mu \omega^{2})}{\mu \omega } & -\frac{jD_{x}D_{z}}{\mu \omega } & 0 & 0 \end{array}\right), \end{array}$$

where $D_{x}=\partial /\partial x$, $D_{y}=\partial /\partial y$, $D_{z}=\partial /\partial z$. Further specific examples in electromagnetism, elasticity and more general fields are reported in [5,10,12,33,34,38].

Here, we assume that the geometry of the problem is invariant along the *z*-direction; thus, without loss of generality, we assume $\psi_{t}=\psi_{t}(x,y,z)=f(x,y)\;{\text{e}}^{-j\alpha_{o}z}$. This yields $\left(\partial /\partial z)\psi_{t}(x,y,z)=-j\alpha_{o}\psi_{t}(x,y,z\right)$, i.e. $\partial /\partial z\to -j\alpha_{o}$ , thus

2.22 $$M\left(\frac{\partial }{\partial z},\frac{\partial }{\partial x}\right)=M\left(-j\alpha_{o},\frac{\partial }{\partial x}\right)=M_{o}+M_{1}\frac{\partial }{\partial x}+M_{2}\frac{\partial^{2}}{\partial x^{2}}+M_{3}\frac{\partial^{3}}{\partial x^{3}}\cdots .$$

Taking into account (2.19), the number of non-null terms at the second member of (2.22) depends on $\Gamma_{t}$ and thus it is three, i.e. $M_{m}=0$ for m>2. The explicit expressions of the matrices $M_{m}$ are defined by the problem under investigation and, in a general electromagnetic medium, the matrices $M_{m}$ are of dimension 4. In an isotropic medium, from (2.21), we have

2.23 $$\begin{array}{rl} & M_{o}=\left(\begin{array}{cccc} 0 & 0 & 0 & \frac{j(-\alpha_{0}^{2}+\varepsilon \mu \omega^{2})}{\varepsilon \omega }\\ 0 & 0 & -j\mu \omega & 0\\ 0 & -\frac{j(-\alpha_{0}^{2}+\varepsilon \mu \omega^{2})}{\mu \omega } & 0 & 0\\ j\varepsilon \omega & 0 & 0 & 0 \end{array}\right)\\ \;\text{and}\; & M_{1}=\left(\begin{array}{cccc} 0 & 0 & -\frac{\alpha_{0}}{\varepsilon \omega } & 0\\ 0 & 0 & 0 & \frac{\alpha_{0}}{\varepsilon \omega }\\ \frac{\alpha_{0}}{\mu \omega } & 0 & 0 & 0\\ 0 & -\frac{\alpha_{0}}{\mu \omega } & 0 & 0 \end{array}\right),\;M_{2}=\left(\begin{array}{cccc} 0 & 0 & 0 & 0\\ 0 & 0 & -\frac{j}{\varepsilon \omega } & 0\\ 0 & 0 & 0 & 0\\ \frac{j}{\mu \omega } & 0 & 0 & 0 \end{array}\right), \end{array}\}$$

where we have omitted the dependence on $-j\alpha_{o}$.

The explicit expression of M (2.19) for a general arbitrary linear medium in electromagnetic applications is reported in [3], while in appendix A we report the anisotropic case. For readability, in the following, we will develop explicit expressions in isotropic media even if the theory and the procedure are completely valid for the general case. As shown in [12,33,34,37], the transverse equations are very useful (independently from the application of §3) to deduce the WH equation in stratified media with discontinuity at the interfaces.

### (a) The eigenvalues and the eigenvectors of M in the spectral domain

By applying the Fourier transform along the x-direction to (2.18) with (2.22)–(2.23) ($M_{m}=0,\;m>2$) in the absence of a source, we obtain an ordinary vector first-order differential equation

2.24 $$-\frac{\text{d}}{\text{d}y}\Psi_{t}(\eta )=M(\eta )\cdot \Psi_{t}(\eta ),$$

where $\psi_{t}(x)=(1/2\pi )\int_{-\infty }^{\infty }\Psi_{t}(\eta )\;{\text{e}}^{-j\eta x}\;\text{d}\eta$ (notation with omission of the y,z dependence) and

2.25 $$M(\eta )=M(-j\alpha_{o},-j\eta )=M_{o}-j\eta M_{1}-\eta^{2}M_{2},$$

where $\partial /\partial z\to -j\alpha_{o}$ for the presence of the field factor ${\text{e}}^{-j\alpha_{o}\;z}$ (see also the comment before (2.22)) and ∂/∂x→−jη for the property of Fourier transforms.

Now let us investigate the properties of the eigenvalue problem (2.26) associated with the differential problem

2.26 $$M(\eta )\cdot u_{i}(\eta )=\lambda_{i}(\eta )u_{i}(\eta ).$$

We anticipate that the eigenvalues $\lambda_{i}$ and the eigenvectors $u_{i}(\eta )$ (i=1…4) of the matrix M(η) (2.26) in rectangular-shaped regions will play a fundamental role in getting the functional equations of an angular region as a solution to the differential problem.

In the presence of a passive medium, we observe that two eigenvalues (say $\lambda_{1},\;\lambda_{2}$) present the non-negative real part and the other two eigenvalues (say $\lambda_{3},\;\lambda_{4}$) present the non-positive real part. While $\lambda_{1},\;\lambda_{2}$ are related to progressive waves, $\lambda_{3},\;\lambda_{4}$ are associated with regressive waves. In this framework, we associate the direction of propagation with attenuation phenomena, while we allow the phase variation to be free of any constraints to also model left-handed materials.

The eigenvalues of the matrix M(η) are

2.27 $$\lambda_{1}=j\;\xi_{1}(\eta ),\;\lambda_{2}=j\;\xi_{2}(\eta ),\;\lambda_{3}=-j\;\xi_{3}(\eta )\;\text{and}\;\lambda_{4}=-j\;\xi_{4}(\eta ).$$

In a medium with reflection symmetry, we have $\xi_{3,4}(\eta )=\xi_{1,2}(\eta )$. For simplicity and to get explicit simple expressions, let us consider homogeneous isotropic lossy media (see the extension to anisotropic media in appendix A). For these media, we have

2.28 $$\xi_{i}(\eta )=\xi (\eta )=\sqrt{\tau_{o}^{2}-\eta^{2}},\;i=1,2,3,4,$$

where $\tau_{o}=\sqrt{k^{2}-\alpha_{o}^{2}}$ with $\text{Im}[\tau_{o}]<0$ and $k=\omega \sqrt{\varepsilon \mu }$ is the propagation constant with Im[k]<0 (normally Re[k]>0; otherwise, Re[k]<0 in left-handed materials). Since $k^{2}=k_{x}^{2}+k_{y}^{2}+k_{z}^{2}=\eta^{2}+\xi^{2}+\alpha_{o}^{2}$, ξ(η) is a multivalued function of *η*. In the following, we assume as a proper sheet of ξ(η) that with $\xi (0)=\tau_{o}$ and as branch lines the classical line Im[ξ(η)]=0 (see ch. 5.3b in [37]) or the vertical line $\left(\text{Re}[\eta ]=\text{Re}[\tau_{o}],\;\text{Im}[\eta ]<\text{Im}[\tau_{o}]\right)$.

In isotropic media, according to (2.27) and (2.28), the eigenvalues are $\lambda_{1,2}=-\lambda_{3,4}=j\xi (\eta )$. The eigenvectors $u_{i}(\eta )=u_{i}$ corresponding to $\lambda_{i},\;i=1,2,3,4$ are

2.29 $$u_{1}=\left|\begin{array}{c} \frac{\tau_{o}^{2}}{\omega \varepsilon \xi }\\ -\frac{\alpha_{o}\eta }{\omega \varepsilon \xi }\\ 0\\ 1 \end{array}\right|,\;u_{2}=\left|\begin{array}{c} \frac{\alpha_{o}\eta }{\omega \varepsilon \xi }\\ -\frac{\left(\xi^{2}+\alpha_{o}^{2}\right)}{\omega \varepsilon \xi }\\ 1\\ 0 \end{array}\right|,\;u_{3}=\left|\begin{array}{c} -\frac{\tau_{o}^{2}}{\omega \varepsilon \xi }\\ \frac{\alpha_{o}\eta }{\omega \varepsilon \xi }\\ 0\\ 1 \end{array}\right|\;\text{and}\;u_{4}=\left|\begin{array}{c} -\frac{\alpha_{o}\eta }{\omega \varepsilon \xi }\\ \frac{\left(\xi^{2}+\alpha_{o}^{2}\right)}{\omega \varepsilon \xi }\\ 1\\ 0 \end{array}\right|.$$

We also introduce the reciprocal vectors $\nu_{i}(\eta )$ of the eigenvectors $u_{i}(\eta )$ that are the eigenvectors of the transpose of the matrix M(η). The vectors $\nu_{i}(\eta )$ satisfy the bi-orthogonal relations

2.30 $$\nu_{j}\cdot u_{i}=\delta_{ji}$$

or alternatively

2.31 $$1_{t}=u_{1}\nu_{1}+u_{2}\nu_{2}+u_{3}\nu_{3}+u_{4}\nu_{4},$$

where $\delta_{ij}$ is the Kronecker symbol, $1_{t}$ is the identity dyadic such that $1_{t}\cdot M=M\cdot 1_{t}$ and in (2.31) we assume dyadic products.

According to the definition reported in (2.30), we obtain from (2.29) the reciprocal vectors $\nu_{i}(\eta )=\nu_{i}$

2.32 $$\begin{array}{rl} & \nu_{1}=\left|\begin{array}{cccc} \frac{\xi^{2}+\alpha_{o}^{2}}{2\omega \mu \xi } & \frac{\alpha_{0}\eta }{2\omega \mu \xi } & 0 & \frac{1}{2} \end{array}\right|,\;\nu_{2}=\left|\begin{array}{cccc} -\frac{\alpha_{0}\eta }{2\omega \mu \xi } & -\frac{\tau_{o}^{2}}{2\omega \mu \xi } & \frac{1}{2} & 0 \end{array}\right|\\ \; & \nu_{3}=\left|-\begin{array}{cccc} \frac{\xi^{2}+\alpha_{o}^{2}}{2\omega \mu \xi } & -\frac{\alpha_{0}\eta }{2\omega \mu \xi } & 0 & \frac{1}{2} \end{array}\right|,\;\nu_{4}=\left|\begin{array}{cccc} \frac{\alpha_{0}\eta }{2\omega \mu \xi } & \frac{\tau_{o}^{2}}{2\omega \mu \xi } & \frac{1}{2} & 0 \end{array}\right|. \end{array}\}$$

## 3. First-order differential oblique transverse equations for angular regions filled by arbitrary linear homogeneous media

In this section, we introduce the oblique transverse equations using an oblique system of Cartesian axes and applying the properties reported in §2 for rectangular regions. In the following sections, first, we deduce spectral functional equations, then, by imposing boundary conditions, the GWHEs for any arbitrary medium with angular shape [3,4].

**Figure 1.**
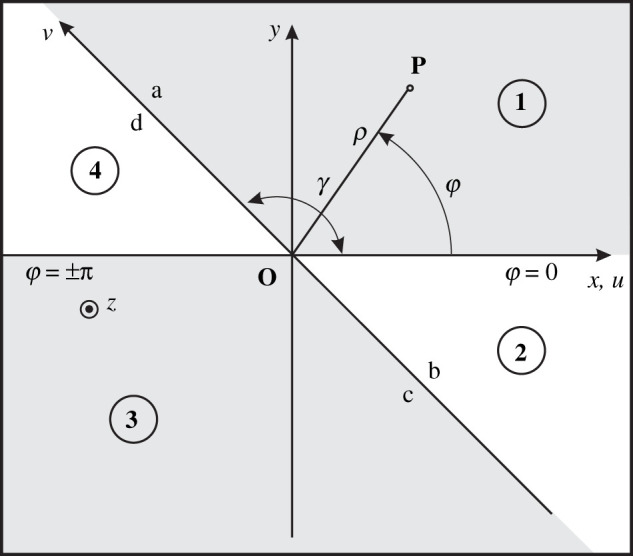
Angular regions and oblique Cartesian coordinates. The figure reports the x,y,z Cartesian coordinates and ρ,φ,z cylindrical coordinates, which are useful to define the oblique Cartesian coordinate system u,v,z with reference to the angular region 1:0<φ<γ with 0<γ<π. In the figure, the space is divided into four angular regions delimited by φ=γ and the face boundaries are labelled a–d.

With reference to figure 1, where angular regions are defined through the angle *γ* (0<γ<π), let us introduce the oblique Cartesian coordinates u,v,z in terms of the Cartesian coordinates x,y,z

3.1 $$u=x-y\cot\gamma ,\;v=\frac{y}{\sin\gamma }\;\text{or}\;x=u+v\cos\gamma ,\;y=v\sin\gamma ,$$

with partial derivatives

3.2 $$\begin{array}{rl} & \frac{\partial }{\partial x}=\frac{\partial u}{\partial x}\frac{\partial }{\partial u}+\frac{\partial v}{\partial x}\frac{\partial }{\partial v}=\frac{\partial }{\partial u},\\ & \frac{\partial }{\partial y}=\frac{\partial u}{\partial y}\frac{\partial }{\partial u}+\frac{\partial v}{\partial y}\frac{\partial }{\partial v}=-\cot\gamma \frac{\partial }{\partial u}+\frac{1}{\sin\gamma }\frac{\partial }{\partial v},\\ & \frac{\partial }{\partial u}=\frac{\partial x}{\partial u}\frac{\partial }{\partial x}+\frac{\partial y}{\partial u}\frac{\partial }{\partial y}=\frac{\partial }{\partial x}\\ \;\text{and}\; & \frac{\partial }{\partial v}=\frac{\partial x}{\partial v}\frac{\partial }{\partial x}+\frac{\partial y}{\partial v}\frac{\partial }{\partial y}=\cos\gamma \frac{\partial }{\partial x}+\sin\gamma \frac{\partial }{\partial y}. \end{array}\}$$

In the following, we consider the system of transverse (with respect to *y*) equations of dimension 4 for an electromagnetic problem with invariant geometry along the *z*-direction (i.e. ${\text{e}}^{-j\alpha_{o}z}$ field dependence) in an arbitrary homogeneous linear medium (see §2, in particular (2.18) with (2.22) and (2.23)):

3.3 $$-\frac{\partial }{\partial y}\psi_{t}=M\left(-j\alpha_{o},\frac{\partial }{\partial x}\right)\cdot \psi_{t}=\left(M_{o}+M_{1}\frac{\partial }{\partial x}+M_{2}\frac{\partial^{2}}{\partial x^{2}}\right)\cdot \psi_{t}.$$

Substituting (3.2), in particular ∂/∂x=∂/∂u and ∂/∂y=−cot⁡γ(∂/∂u)+(1/sin⁡γ)(∂/∂v), into (3.3), we obtain

3.4 $$-\frac{\partial }{\partial v}\psi_{t}=M_{e}\left(-j\alpha_{o},\frac{\partial }{\partial u}\right)\cdot \psi_{t}=\left(M_{eo}+M_{e1}\frac{\partial }{\partial u}+M_{e2}\frac{\partial^{2}}{\partial u^{2}}\right)\cdot \psi_{t},$$

where

3.5 $$M_{eo}=M_{o}\sin\gamma ,\;M_{e1}=M_{1}\sin\gamma -I_{t}\cos\gamma ,\;M_{e2}=M_{2}\sin\gamma .$$

For the sake of simplicity and in order to get simple explicit expressions, let us consider a homogeneous isotropic medium, even if the procedure is general and applicable to arbitrary linear media (definitions for the anisotropic case are reported in appendix A). For isotropic media, we have

3.6 $$\begin{array}{r} \begin{array}{rl} & M_{eo}=\left(\begin{array}{cccc} 0 & 0 & 0 & \frac{j(-\alpha_{0}^{2}+\varepsilon \mu \omega^{2})\sin\gamma }{\varepsilon \omega }\\ 0 & 0 & -j\mu \omega \sin\gamma & 0\\ 0 & -\frac{j(-\alpha_{0}^{2}+\varepsilon \mu \omega^{2})\sin\gamma }{\mu \omega } & 0 & 0\\ j\varepsilon \omega \sin\gamma & 0 & 0 & 0 \end{array}\right),\\ & M_{e1}=\left(\begin{array}{cccc} -\cos\gamma & 0 & -\frac{\alpha_{0}\sin\gamma }{\varepsilon \omega } & 0\\ 0 & -\cos\gamma & 0 & \frac{\alpha_{0}\sin\gamma }{\varepsilon \omega }\\ \frac{\alpha_{0}\sin\gamma }{\mu \omega } & 0 & -\cos\gamma & 0\\ 0 & -\frac{\alpha_{0}\sin\gamma }{\mu \omega } & 0 & -\cos\gamma \end{array}\right)\\ \;\text{and}\; & M_{e2}=\left(\begin{array}{cccc} 0 & 0 & 0 & 0\\ 0 & 0 & -\frac{j\sin\gamma }{\varepsilon \omega } & 0\\ 0 & 0 & 0 & 0\\ \frac{j\sin\gamma }{\mu \omega } & 0 & 0 & 0 \end{array}\right). \end{array}\}. \end{array}$$

By applying the Fourier transform along the x=u direction to (3.4) (i.e. $\psi_{t}(x)=(1/2\pi )\int_{-\infty }^{\infty }\Psi_{t}(\eta ){\text{e}}^{-j\eta x}\;\text{d}\eta$ with notation omitting v,z dependence), we obtain

3.7 $$-\frac{\text{d}}{\text{d}v}\Psi_{t}(\eta )=M_{e}(\gamma ,\eta )\cdot \Psi_{t}(\eta ),$$

with

3.8 $$M_{e}(\gamma ,\eta )=M_{e}(-j\alpha_{o},-j\eta )=M_{eo}-j\eta M_{e1}-\eta^{2}M_{e2},$$

since ∂/∂u=∂/∂x→−jη.

### (a) Link between eigenvalues of M(η) and $M_{e}(\gamma ,\eta )$

In oblique coordinates, the solution of (3.7) is related to the eigenvalue problem

3.9 $$M_{e}(\gamma ,\eta )\cdot u_{ei}(\gamma ,\eta )=\lambda_{ei}(\gamma ,\eta )u_{ei}(\gamma ,\eta ),$$

where $\lambda_{ei}$ and $u_{ei}(\eta )$ (i=1…n) are, respectively, the eigenvalues and the eigenvectors of the matrix $M_{e}(\gamma ,\eta )$ of dimension n=4 in our application. Using (3.5) and (3.8), equation (3.9) becomes

3.10 $$\left(M_{o}\sin\gamma -j\eta M_{1}\sin\gamma -\eta^{2}M_{2}\sin\gamma )\cdot u_{ei}(\gamma ,\eta )=(\lambda_{ei}(\gamma ,\eta )-j\eta \cos\gamma )u_{ei}(\gamma ,\eta \right)$$

and thus

3.11 $$M(\eta )\cdot u_{ei}(\gamma ,\eta )=\left(\frac{\lambda_{ei}(\gamma ,\eta )-j\eta \cos\gamma }{\sin\gamma }\right)u_{ei}(\gamma ,\eta ).$$

Comparing (3.11) with (2.26), we observe the relation among the eigenvalues and the eigenvectors of the two problems. The two problems defined by the matrices M(η) and $M_{e}(\gamma ,\eta )$ have the same eigenvectors

3.12 $$u_{ei}(\gamma ,\eta )=u_{i}(\eta ),$$

and thus the same reciprocal vectors and related eigenvalues

3.13 $$\frac{\lambda_{ei}(\gamma ,\eta )-j\eta \cos\gamma }{\sin\gamma }=\lambda_{i}(\eta ).$$

Since $M_{e}(\gamma ,\eta )$ and M(η) have the same eigenvectors (3.12) and the eigenvectors of M(η) are $u_{i}(\eta )$ reported in (2.29), we note the important property that the eigenvectors of $M_{e}(\gamma ,\eta )$ do not depend on the aperture angle *γ* (figure 1). From (3.13), the eigenvalues $\lambda_{ei}$ of $M_{e}(\gamma ,\eta )$ can be rewritten using the notation (2.27)

3.14 $$\begin{array}{rl} & \lambda_{e1}(\gamma ,\eta )=j(\eta \cos\gamma +\sin\gamma \xi_{1}(\eta )),\\ & \lambda_{e2}(\gamma ,\eta )=j(\eta \cos\gamma +\sin\gamma \xi_{2}(\eta )),\\ & \lambda_{e3}(\gamma ,\eta )=j(\eta \cos\gamma -\sin\gamma \xi_{3}(\eta ))\\ \;\text{and}\; & \lambda_{e4}(\gamma ,\eta )=j(\eta \cos\gamma -\sin\gamma \xi_{4}(\eta )), \end{array}\},$$

where $\lambda_{e1},\lambda_{e2}$ ($\lambda_{e3},\lambda_{e4}$) are related to progressive (regressive) waves.

For what concerns the specific case of electromagnetic applications with a homogeneous isotropic medium in angular regions, the eigenvalues of the matrix $M_{e}(\gamma ,\eta )$ are

3.15 $$\begin{array}{rl} & \lambda_{e1}=\lambda_{e2}=j\eta \cos\gamma +j\sqrt{{\tau_{o}}^{2}-\eta^{2}}\sin\gamma \\ \;\text{and}\; & \lambda_{e3}=\lambda_{e4}=j\eta \cos\gamma -j\sqrt{{\tau_{o}}^{2}-\eta^{2}}\sin\gamma , \end{array}\}$$

where k is the propagation constant, $\tau_{o}=\sqrt{k^{2}-\alpha_{o}^{2}}$ and $\xi =\xi (\eta )=\sqrt{\tau_{o}^{2}-\eta^{2}}$ (2.28) is a multivalued function as discussed in §2a. Note that, also in the isotropic angular geometries, two independent eigenvectors $u_{1},u_{2}$ ($u_{3},u_{4}$) (2.29) correspond to the two equal eigenvalues $\lambda_{e1}=\lambda_{e2}$ ($\lambda_{e3}=\lambda_{e4}$) as reported in (3.15).

## 4. Solution of the oblique transverse equations

In order to present the general solution procedure, in the following, we consider a system of oblique transverse equations (3.4) of dimension 4 with matrix operator $M_{e}(-j\alpha_{o},\partial /\partial u)$ with three non-null terms ($M_{o},M_{1},M_{2}$) for a problem with invariant geometry along the *z*-direction. This framework is appropriate for electromagnetic applications in arbitrary linear media and it will be explicitly developed for particular problems in §5. In this section, we obtain, as a general solution, the spectral functional equations for the four angular regions as identified in figure 1. The four angular regions present the same equation (3.4) but with different matrices $M_{eo},M_{e1},M_{e2}$ depending on the medium as well as the aperture angle *γ*.

Let us introduce the Laplace transforms (notation omitting *z* dependence)

4.1 $${\tilde{\psi }}_{t}(\eta ,v)=\int_{0}^{\infty }\text{e}{\;}^{j\eta \;u}\psi_{t}(u,v)\;\text{d}u$$

for regions 1,2 and

4.2 $${\tilde{\psi }}_{t}(\eta ,v)=\int_{-\infty }^{0}{\text{e}}^{j\eta \;u}\psi_{t}(u,v)\;\text{d}u$$

for regions 3,4.

The Laplace transforms applied to (3.4) yields

4.3 $$-\frac{\text{d}}{\text{d}v}{\tilde{\psi }}_{t}(\eta ,v)=M_{e}(\gamma ,\eta )\cdot {\tilde{\psi }}_{t}(\eta ,v)+\psi_{s}(v),$$

with

4.4 $$M_{e}(\gamma ,\eta )=M_{e}(-j\alpha_{o},-j\eta )=M_{eo}-j\eta M_{e1}-\eta^{2}M_{e2}.$$

Note that (4.4) and (3.8) share the same symbol and explicit mathematical expression; however, the first is related to a Fourier transform while the second is related to a Laplace transform, thus obviously they have the same eigenvalues and eigenvectors. The term $\psi_{s}(v)$ is obtained from the derivative property of the Laplace transform (initial conditions) and for each angular region we obtain a different expression. In particular, we indicate with $\psi_{as}(v)$ the value of $\psi_{s}(v)$ on face a (see figure 1, $0\leq v<+\infty ,u=0_{+}$), with $\psi_{bs}(v)$ the value of $\psi_{s}(v)$ on face b ($-\infty \leq v<0,u=0_{+}$), with $\psi_{cs}(v)$ the value of $\psi_{s}(v)$ on face c ($-\infty \leq v<0,u=0_{-}$) and with $\psi_{ds}(v)$ the value of $\psi_{s}(v)$ on face d ($0\leq v<+\infty ,u=0_{-}$).

Since (4.3) is a system of four ordinary differential equations of first order with constant coefficients in a semi-infinite interval, we mainly have two methods for its solution: (i) apply the dyadic Green’s function procedure in the *v* domain and (ii) apply the Laplace transform in *v* that yields a linear system of four algebraic equations from which one can write down the general solution in terms of eigenvalues and eigenfunctions. We note that both methods are effective and in particular the second method is more useful for representing the spectral solution in each point of the considered angular region. However, it initially introduces complex functions of two variables. As proposed in the following subsections, we prefer the first method because, in this way, we get the functional equations of the angular regions that directly involve complex functions of one variable.

Using the concept of non-standard Laplace transforms (see §1.4 of [5]), the validity of (4.3) and (4.4) in the absence of sources is extended to the total fields in the presence of plane-wave sources or in general of sources located at infinity.

With reference to figure 1, let us now describe the four angular regions in detail. The selection of four angular regions as in figure 1 related to a unique aperture angle *γ* does not limit the applicability of the method. In fact, all the equations (once derived) can be used with a different appropriate aperture angle, just replacing *γ* with the proper value. The purpose of deriving the functional equations with a unique *γ* is related to the fact that we formulate and solve the angular region problems by analysing once and for all the matrix operator $M_{e}(\gamma ,\eta )$ (4.4). We recall also that the imposition of boundary conditions and media for each region will be made only while examining a practical problem and that it yields GWHEs.

### (a) Region 1: u>0,v>0

With reference to figure 1, for what concerns region 1 (u>0,v>0), (4.3) holds with

4.5 $$\psi_{s}(v)=\psi_{as}(v)=-M_{e1}\cdot \psi_{t}(0_{+},v)+j\eta M_{e2}\cdot \psi_{t}(0_{+},v)-M_{e2}\cdot {\frac{\partial }{\partial u}\psi_{t}(u,v)|}_{u=0+}.$$

Equation (4.3) is a system of differential equations of first order (of dimension 4 in our electromagnetic assumption), whose solution ${\tilde{\psi }}_{t}(\eta ,v)$ is obtainable as the sum of a particular integral ${\tilde{\psi }}_{p}(\eta ,v)$ with the general solution of the homogeneous equation ${\tilde{\psi }}_{o}(\eta ,v)$

4.6 $${\tilde{\psi }}_{t}(\eta ,v)={\tilde{\psi }}_{o}(\eta ,v)+{\tilde{\psi }}_{p}(\eta ,v).$$

The solution of the homogeneous equation must satisfy

4.7 $$-\frac{\text{d}}{\text{d}v}{\tilde{\psi }}_{o}(\eta ,v)=M_{e}(\gamma ,\eta )\cdot {\tilde{\psi }}_{o}(\eta ,v).$$

Considering the solution form ${\tilde{\psi }}_{o}(\eta ,v)=C\;{\text{e}}^{-\lambda (\gamma ,\eta )v}u(\gamma )$, the most general solution is

4.8 $$\begin{array}{rl} {\tilde{\psi }}_{o}(\eta ,v) & =C_{1}\;{\text{e}}^{-\lambda_{e1}(\gamma )v}u_{1}(\eta )+C_{2}\;{\text{e}}^{-\lambda_{e2}(\gamma ,\eta )v}u_{2}(\eta )\\ & \;+C_{3}\;{\text{e}}^{-\lambda_{e3}(\gamma ,\eta )v}u_{3}(\eta )+C_{4}\;{\text{e}}^{-\lambda_{e4}(\gamma ,\eta )v}u_{4}(\eta ), \end{array}$$

where $\lambda_{ei}$ and $u_{ei}\equiv u_{i}$ (i=1,2,3,4) are the eigenvalues and the eigenvectors of the matrix $M_{e}(\gamma ,\eta )$, respectively reported in (3.14) and (3.12).

In the presence of a passive medium, we recall that two eigenvalues (say $\lambda_{1},\;\lambda_{2}$) present a non-negative real part and the other two eigenvalues (say $\lambda_{3},\;\lambda_{4}$) present a non-positive real part. From (3.14), we note that $\lambda_{e1},\lambda_{e2}$ model progressive waves along a positive *v* direction, while $\lambda_{e1},\lambda_{e2}$ model regressive waves.

The evaluation of the particular integral of (4.3)

4.9 $${\tilde{\psi }}_{p}(\eta ,v)=-\int_{0}^{\infty }G(v,v^{\prime })\cdot \psi_{s}(v^{\prime })\;\text{d}v^{\prime }$$

requires the dyadic Green’s function $G(v,v^{\prime })$ of (4.3), i.e. the solution of

4.10 $$\left(\frac{\text{d}}{\text{d}v}+M_{e}(\gamma ,\eta )\right)\cdot G(v,v^{\prime })=\delta (v-v^{\prime })1_{t},$$

with the boundary condition of the problem: in this case, those of region 1 (u>0,v>0). Note that $1_{t}$ is the identity dyadic of dimension 4 in our assumption (2.31).

An original method to get the particular solution is reported in [3,33,34]. While in [33,34] the method is applied to arbitrary stratified regions with appropriate boundary conditions, in this paper, we apply a slightly different method to the simplified structure consisting of an arbitrary indefinite angular region for the solution of (4.10). According to [39], it is possible to build a Green’s function starting from arbitrary solutions of the homogeneous equations without imposing boundary conditions at first. Then, to get the solution to the differential problem with the boundary conditions, the selected form of the particular integral influences the values of the arbitrary coefficients of the homogeneous solutions for the imposition of the boundary conditions. Finally, the sum of the homogeneous solutions with the particular integrals yields the solution to the problem.

We select progressive and regressive waves in an indefinite half-space as homogeneous solutions for building the dyadic Green’s function (see appendix B for the justification and properties of the dyadic Green’s function). In our framework, we avoid imposing the boundary condition at this point, since we want to find functional equations that are free of this constraint. Only, while investigating a practical problem, we will impose boundary conditions on the functional equations (for instance, in region 1 at face φ=0, i.e. u>0, v=0, and face φ=γ, i.e. u=0, v>0), yielding GWHEs of the problem. See §5b for a practical example of the wedge-scattering problem.

By applying this method (appendix B) to the present problem, we obtain the dyadic Green’s function

4.11 $$G(v,v^{\prime })=\{\begin{array}{ll} u_{1}\nu_{1}\;{\text{e}}^{-\lambda_{e1}(\gamma ,\eta )(v-v^{\prime })}+u_{2}\nu_{2}\;{\text{e}}^{-\lambda_{e2}(\gamma ,\eta )(v-v^{\prime })}, & v>v^{\prime },\\ -[u_{3}\nu_{3}\;{\text{e}}^{-\lambda_{e3}(\gamma ,\eta )(v-v^{\prime })}+u_{4}\nu_{4}\;{\text{e}}^{-\lambda_{e4}(\gamma ,\eta )(v-v^{\prime })}], & v<v^{\prime }, \end{array}$$

where $\nu_{i}$ are the reciprocal vectors (2.30) of the eigenvectors $u_{i}$ of $M_{e}(\gamma ,\eta )$ and $\lambda_{ei}$ are the related eigenvalues. Note that $u_{i}\nu_{i}$ in (4.11) are dyadic products.

By substituting (4.8) and (4.9) with (4.11) into (4.6), this yields

4.12 $$\begin{array}{rl} {\tilde{\psi }}_{t}(\eta ,v) & =C_{1}u_{1}\;{\text{e}}^{-\lambda_{e1}(\gamma ,\eta )v}+C_{2}u_{2}\;{\text{e}}^{-\lambda_{e2}(\gamma ,\eta )v}+C_{3}u_{3}\;{\text{e}}^{-\lambda_{e3}(\gamma ,\eta )v}+C_{4}u_{4}\;{\text{e}}^{-\lambda_{e4}(\gamma ,\eta )v}\\ & \;-u_{1}\nu_{1}\cdot \int_{0}^{v}{\text{e}}^{-\lambda_{e1}(\gamma ,\eta )(v-v^{\prime })}\psi_{as}(v^{\prime })\;\text{d}v^{\prime }-u_{2}\nu_{2}\cdot \int_{0}^{v}\;{\text{e}}^{-\lambda_{e2}(\gamma ,\eta )(v-v^{\prime })}\psi_{as}(v^{\prime })\;\text{d}v^{\prime }\\ & \;+u_{3}\nu_{3}\cdot \int_{v}^{\infty }{\text{e}}^{-\lambda_{e3}(\gamma ,\eta )(v-v^{\prime })}\psi_{as}(v^{\prime })\;\text{d}v^{\prime }+u_{4}\nu_{4}\cdot \int_{v}^{\infty }{\text{e}}^{-\lambda_{e4}(\gamma ,\eta )(v-v^{\prime })}\psi_{as}(v^{\prime })\;\text{d}v^{\prime }. \end{array}$$

Looking at the asymptotic behaviour of (4.12) for v→+∞ we have that only the terms $C_{3}u_{3}\;{\text{e}}^{-\lambda_{e3}v}+C_{4}u_{4}\;{\text{e}}^{-\lambda_{e4}v}$ are divergent. For this reason, we assume $C_{3}=C_{4}=0$. Note, in particular, the vanishing of the last two integral terms as v→+∞.

Setting v=0 in (4.12), we have

4.13 $${\tilde{\psi }}_{t}(\eta ,0)=C_{1}u_{1}+C_{2}u_{2}+u_{3}\nu_{3}\cdot \int_{0}^{\infty }{\text{e}}^{\lambda_{e3}(\gamma ,\eta )v^{\prime }}\psi_{as}(v^{\prime })\;\text{d}v^{\prime }+u_{4}\nu_{4}\cdot \int_{0}^{\infty }{\text{e}}^{\lambda_{e4}(\gamma ,\eta )v^{\prime }}\psi_{as}(v^{\prime })\;\text{d}v^{\prime }.$$

Multiplying (4.13) by $\nu_{i}(\eta )=\nu_{i}$ for i=1…4, we obtain

4.14 $$\begin{array}{rl} & \nu_{1}(\eta )\cdot {\tilde{\psi }}_{t}(\eta ,0)=C_{1},\\ & \nu_{2}(\eta )\cdot {\tilde{\psi }}_{t}(\eta ,0)=C_{2},\\ & \nu_{3}(\eta )\cdot {\tilde{\psi }}_{t}(\eta ,0)=\nu_{3}(\eta )\cdot {\overset{⌣}{\psi }}_{as}(-j\lambda_{e3}(\gamma ,\eta ))\\ \;\text{and}\; & \nu_{4}(\eta )\cdot {\tilde{\psi }}_{t}(\eta ,0)=\nu_{4}(\eta )\cdot {\overset{⌣}{\psi }}_{as}(-j\lambda_{e4}(\gamma ,\eta )) \end{array}\}$$

owing to the property of the reciprocal vectors (2.30) and where ${\overset{⌣}{\psi }}_{as}(\chi )$ is the Laplace transform in *v* along face a (v=ρ in cylindrical coordinates)

4.15 $${\overset{⌣}{\psi }}_{as}(\chi )=\int_{0}^{\infty }\;{\text{e}}^{j\chi v}\psi_{as}(v)\;\text{d}v.$$

The last two equations of (4.14) can be rewritten in the form

4.16 $$\nu_{3}(\eta )\cdot {\tilde{\psi }}_{t}(\eta ,0)=\nu_{3}(\eta )\cdot {\overset{⌣}{\psi }}_{as}(-m_{a1}(\gamma ,\eta ))$$

and

4.17 $$\nu_{4}(\eta )\cdot {\tilde{\psi }}_{t}(\eta ,0)=\nu_{4}(\eta )\cdot {\overset{⌣}{\psi }}_{as}(-m_{a2}(\gamma ,\eta )),$$

with

4.18 $$\begin{array}{rl} & m_{a1}(\gamma ,\eta )=j\lambda_{e3}(\gamma ,\eta )=-\eta \cos\gamma +\xi_{1}\sin\gamma \\ \;\text{and}\; & m_{a2}(\gamma ,\eta )=j\lambda_{e4}(\gamma ,\eta )=-\eta \cos\gamma +\xi_{2}\sin\gamma . \end{array}\}$$

While the first two equations of (4.14) relate the unknowns $C_{1}$ and $C_{2}$ to the Laplace transform ${\tilde{\psi }}_{t}(\eta ,0)$ evaluated in the lower face of the angular region (u>0, v=0), the last two equations of (4.14) provide two important functional equations that relate the Laplace transforms of combinations of the field components on the boundaries of the angular region 1, i.e. u>0, v=0 and u=0, v>0 (face a) in figure 1.

These functional equations are the starting point to define the GWHEs of region 1. They are valid for any linear medium filling the region and are independent of any boundary conditions surrounding the region.

For example, and for simplicity, the explicit forms of (4.16) and (4.17) are reported in §5 for isotropic media where $\xi_{i}(\eta )=\xi (\eta )=\sqrt{\tau_{o}^{2}-\eta^{2}}$ (see the definition of the multivalued function ξ(η) in §3a).

These functional equations are equivalent to (3.3.57) and (3.3.58) in [5], where a completely different method has been applied for the derivation. In fact, in ch. 3 of [5], the equations are obtained from the second-order differential formulation for electromagnetic applications (wave equation). The theory is developed for an isotropic medium and cumbersome symmetry properties have been used to develop the equations for the other angular regions with respect to region 1.

In the present work, the theory is more general and is applicable to any arbitrary electromagnetic media and extendable to different physics. In particular, the equations for the other regions with respect to region 1 are easily derived as in the following subsections.

### (b) Region 2: u>0,v<0

With reference to figure 1, following the procedure reported for region 1 in §4a, we develop the solution for region 2 (u>0,v<0). The problem shows the same equation (4.3) with

4.19 $$\psi_{s}(v)=\psi_{bs}(v)=-M_{e1}\cdot \psi_{t}(0_{+},v)+j\eta M_{e2}\cdot \psi_{t}(0_{+},v)-M_{e2}\cdot {\frac{\partial }{\partial u}\psi_{t}(u,v)|}_{u=0+}.$$

Note the different geometrical support of (4.19) with respect to (4.5), i.e. for region 2 v<0 while for region 1 v>0. As per region 1, the solution of (4.3) is obtained as a combination of the homogeneous solution and the particular integral; see (4.6). We note that the particular integral depends on (4.19), while the homogeneous solution depends on the expressions of eigenvalues $\lambda_{ei}(\gamma ,\eta )$ and eigenvectors $u_{i}(\eta )$ of $M_{e}(\gamma ,\eta )$ (4.4) that are the same as for region 1, except for their dependence on the physical constitutive parameters of region 2 that may be inhomogeneous with respect to region 1.

Once the expression of the dyadic Green’s function specialized for region 2 has been obtained, we get

4.20 $$\begin{array}{rl} {\tilde{\psi }}_{t}(\eta ,v) & =C_{1}u_{1}\;{\text{e}}^{-\lambda_{e1}(\gamma ,\eta )v}+C_{2}u_{2}\;{\text{e}}^{-\lambda_{e2}(\gamma ,\eta )v}+C_{3}u_{3}\;{\text{e}}^{-\lambda_{e3}(\gamma ,\eta )v}+C_{4}u_{4}\;{\text{e}}^{-\lambda_{e4}(\gamma ,\eta )v}\\ & \;-u_{1}\nu_{1}\cdot \int_{-\infty }^{v}{\text{e}}^{-\lambda_{e1}(\gamma ,\eta )(v-v^{\prime })}\psi_{bs}(v^{\prime })\;\text{d}v^{\prime }-u_{2}\nu_{2}\cdot \int_{-\infty }^{v}{\text{e}}^{-\lambda_{e2}(\gamma ,\eta )(v-v^{\prime })}\psi_{bs}(v^{\prime })\;\text{d}v^{\prime }\\ & \;+u_{3}\nu_{3}\cdot \int_{v}^{0}{\text{e}}^{-\lambda_{e3}(\gamma ,\eta )(v-v^{\prime })}\psi_{bs}(v^{\prime })\;\text{d}v^{\prime }+u_{4}\nu_{4}\cdot \int_{v}^{0}{\text{e}}^{-\lambda_{e4}(\gamma ,\eta )(v-v^{\prime })}\psi_{bs}(v^{\prime })\;\text{d}v^{\prime }, \end{array}$$

where $\lambda_{ei}$ and $u_{i}$ are reported in (3.14) and (3.12).

Looking at the asymptotic behaviour of (4.20) for v→−∞, we have that only the terms $C_{1}u_{1}\;{\text{e}}^{-\lambda_{e1}v}+C_{2}u_{2}\;{\text{e}}^{-\lambda_{e2}v}$ are divergent. For this reason, we assume $C_{1}=C_{2}=0$. Note, in particular, the vanishing of the first two integral terms as v→−∞.

Assuming v=0 in (4.20), we have

4.21 $$\begin{array}{rl} {\tilde{\psi }}_{t}(\eta ,0) & =C_{3}u_{3}+C_{4}u_{4}-u_{1}\nu_{1}\int_{-\infty }^{0}{\text{e}}^{-\lambda_{e1}(\gamma ,\eta )(-v^{\prime })}\psi_{bs}(v^{\prime })\;\text{d}v^{\prime }-u_{2}\nu_{2}\int_{-\infty }^{0}{\text{e}}^{-\lambda_{e2}(\gamma ,\eta )(-v^{\prime })}\psi_{bs}(v^{\prime })\;\text{d}v^{\prime }. \end{array}$$

Multiplying (4.21) by $\nu_{i}(\eta )=\nu_{i}$ for i=1…4, we obtain

4.22 $$\begin{array}{rl} & \nu_{3}(\eta )\cdot {\tilde{\psi }}_{t}(\eta ,0)=C_{3},\\ & \nu_{4}(\eta )\cdot {\tilde{\psi }}_{t}(\eta ,0)=C_{4},\\ & \nu_{1}(\eta )\cdot {\tilde{\psi }}_{t}(\eta ,0)=-\nu_{1}(\eta )\cdot {\overset{⌣}{\psi }}_{bs}(j\lambda_{e1}(\gamma ,\eta ))\\ \;\text{and}\; & \nu_{2}(\eta )\cdot {\tilde{\psi }}_{t}(\eta ,0)=-\nu_{2}(\eta )\cdot {\overset{⌣}{\psi }}_{bs}(j\lambda_{e2}(\gamma ,\eta )), \end{array}\},$$

where

4.23 $${\overset{⌣}{\psi }}_{bs}(\chi )=\int_{-\infty }^{0}{\text{e}}^{-j\chi v}\psi_{bs}(v)\;\text{d}v=\int_{0}^{\infty }{\text{e}}^{j\chi \rho }\psi_{bs}(-\rho )\;\text{d}\rho$$

is the left Laplace transform of $\psi_{bs}(v)$ in *v* along face b (figure 1) or the Laplace transform in ρ of $\psi_{bs}(-\rho )$ in cylindrical coordinates (ρ,φ,z).

As stated for region 1, in media with reflection symmetry ($\xi_{3,4}(\eta )=\xi_{1,2}(\eta )$), the last two equations of (4.22) can be rewritten in the form

4.24 $$\nu_{1}(\eta )\cdot {\tilde{\psi }}_{t}(\eta ,0)=-\nu_{1}(\eta )\cdot {\overset{⌣}{\psi }}_{bs}(-m_{b1}(\gamma ,\eta ))$$

and

4.25 $$\nu_{2}(\eta )\cdot {\tilde{\psi }}_{t}(\eta ,0)=-\nu_{2}(\eta )\cdot {\overset{⌣}{\psi }}_{bs}(-m_{b2}(\gamma ,\eta )),$$

with

4.26 $$\begin{array}{rl} & m_{b1}(\gamma ,\eta )=-j\lambda_{e1}(\gamma ,\eta )=\eta \cos\gamma +\xi_{1}\sin\gamma \\ \;\text{and}\; & m_{b2}(\gamma ,\eta )=-j\lambda_{e2}(\gamma ,\eta )=\eta \cos\gamma +\xi_{2}\sin\gamma . \end{array}\}$$

While the first two equations of (4.22) relate the unknowns $C_{3}$ and $C_{4}$ to the Laplace transform ${\tilde{\psi }}_{t}(\eta ,0)$ evaluated at the face of the angular region (u>0, v=0), the last two equations of (4.22) provide two important functional equations that relate the Laplace transforms of combinations of field components on the boundaries of the angular region 2, i.e. u>0, v=0 and u=0, v<0 (face b) in figure 1. These functional equations are the starting point to define the GWHEs of region 2. As stated for region 1, they are valid for any linear medium filling the region and are independent of any boundary conditions surrounding the region. They agree with those proposed in ch. 3 of [5] in the case of an isotropic medium for electromagnetic applications.

Note that, in view of dealing with scattering problems by wedges (see §5b), the aperture angle of region 2 is usually different from *γ*. This difference modifies the equations only in (4.26) for the dependence on a different aperture angle. We recall that the motivation for deriving the functional equations with a unique *γ* is related to the fact that we formulate and solve the angular region problems by analysing once and for a single matrix operator $M_{e}(\gamma ,\eta )$ (4.4).

### (c) Region 4: u<0,v>0

With reference to figure 1, and following the procedure reported for region 1 in §4a, we develop the solution for region 4 (u<0,v>0). Applying the Laplace transform

4.27 $$\begin{array}{rl} & {\tilde{\psi }}_{t}(\eta ,0)=\int_{-\infty }^{0}{\text{e}}^{j\eta \;u}\psi_{t}(u,0)\;\text{d}u={\tilde{\psi }}_{\pi t}(-\eta ,0)\\ \;\text{and}\; & {\tilde{\psi }}_{\pi t}(\eta ,0)=\int_{0}^{\infty }{\text{e}}^{j\eta u}\psi_{t}(-u,0)\;\text{d}u \end{array}\}$$

to (3.4), the problem in region 4 shows the same equation (4.3)

4.28 $$-\frac{\text{d}}{\text{d}v}{\tilde{\psi }}_{t}=M_{e}(\gamma ,\eta )\cdot {\tilde{\psi }}_{t}+\psi_{s}(v),$$

with $M_{e}(\gamma ,\eta )$ reported in (4.4) and with the different definition of

4.29 $$\psi_{s}(v)=\psi_{ds}(v)=M_{e1}\cdot \psi_{t}(0_{-},v)-j\eta M_{e2}\cdot \psi_{t}(0_{-},v)+M_{e2}\cdot {\frac{\partial }{\partial u}\psi_{t}(u,v)|}_{u=0_{-}},$$

which is related to the derivative property of the Laplace transform (4.27) along face d (figure 1).

The application of the method used for region 1 yields the two functional equations

4.30 $$\nu_{3}(\eta )\cdot {\tilde{\psi }}_{t}(\eta ,0)=\nu_{3}(\eta )\cdot {\overset{⌣}{\psi }}_{ds}(-j\lambda_{e3}(\gamma ,\eta ))$$

and

4.31 $$\nu_{4}(\eta )\cdot {\tilde{\psi }}_{t}(\eta ,0)=\nu_{4}(\eta )\cdot {\overset{⌣}{\psi }}_{ds}(-j\lambda_{e4}(\gamma ,\eta )),$$

where we have defined the Laplace transform

4.32 $${\overset{⌣}{\psi }}_{ds}(\chi )=\int_{0}^{\infty }{\text{e}}^{j\chi v}\psi_{ds}(v)\;\text{d}v.$$

The other difference with respect to the last two equations of (4.14) is the definition of

4.33 $${\tilde{\psi }}_{t}(\eta ,u)=\int_{-\infty }^{0}{\text{e}}^{j\eta u}\psi_{t}(u,v)\;\text{d}u,$$

which is a minus function (left Laplace transform). Changing *η* to −η, we rewrite (4.30) and (4.31) as

4.34 $$\nu_{3}(-\eta )\cdot {\tilde{\psi }}_{\pi t}(\eta ,0)=\nu_{3}(-\eta )\cdot {\overset{⌣}{\psi }}_{ds}(-j\lambda_{e3}(\gamma ,-\eta ))$$

and

4.35 $$\nu_{4}(-\eta )\cdot {\tilde{\psi }}_{\pi t}(\eta ,0)=\nu_{4}(-\eta )\cdot {\overset{⌣}{\psi }}_{ds}(-j\lambda_{e4}(\gamma ,-\eta )),$$

with the plus function (right Laplace transform)

4.36 $${\tilde{\psi }}_{\pi t}(\eta ,0)=\int_{0}^{\infty }{\text{e}}^{j\eta u}\psi_{t}(-u,0)\;\text{d}u.$$

### (d) Region 3: u<0,v<0

As already done for regions 1, 2 and 4, we repeat the procedure. We get the same equation (4.3) with the definition ${\tilde{\psi }}_{t}(\eta ,0)$ (4.27) except for

4.37 $$\psi_{s}(v)=\psi_{cs}(v)=M_{e1}\cdot \psi_{t}(0_{-},v)-j\eta M_{e2}\cdot \psi_{t}(0_{-},v)+M_{e2}\cdot {\frac{\partial }{\partial u}\psi_{t}(u,v)|}_{u=0_{-}},$$

which is related to the derivative property of the Laplace transform (4.27) along face c (figure 1).

This yields the two functional equations

4.38 $$\nu_{1}(\eta )\cdot {\tilde{\psi }}_{t}(\eta ,0)=-\nu_{1}(\eta )\cdot {\overset{⌣}{\psi }}_{cs}(j\lambda_{e1}(\gamma ,\eta ))$$

and

4.39 $$\nu_{2}(\eta )\cdot {\tilde{\psi }}_{t}(\eta ,0)=-\nu_{2}(\eta )\cdot {\overset{⌣}{\psi }}_{cs}(j\lambda_{e2}(\gamma ,\eta )),$$

where we have defined the Laplace transform

4.40 $${\overset{⌣}{\psi }}_{cs}(\chi )=\int_{-\infty }^{0}{\text{e}}^{-j\chi v}\psi_{cs}(v)\;\text{d}v=\int_{0}^{\infty }{\text{e}}^{j\chi \rho }\psi_{cs}(-\rho )\;\text{d}\rho .$$

The other difference with respect to the last two equations of (4.14) is the definition of

4.41 $${\tilde{\psi }}_{t}(\eta ,u)=\int_{-\infty }^{0}{\text{e}}^{j\eta u}\psi_{t}(u,v)\;\text{d}u,$$

which is a minus function (left Laplace transform). Changing *η* to −η, we rewrite (4.38) and (4.39) as

4.42 $$\nu_{1}(-\eta )\cdot {\tilde{\psi }}_{\pi t}(\eta ,0)=-\nu_{1}(-\eta )\cdot {\overset{⌣}{\psi }}_{cs}(j\lambda_{e1}(\gamma ,-\eta ))$$

and

4.43 $$\nu_{2}(-\eta )\cdot {\tilde{\psi }}_{\pi t}(\eta ,0)=-\nu_{2}(-\eta )\cdot {\overset{⌣}{\psi }}_{cs}(j\lambda_{e2}(\gamma ,-\eta )),$$

with the plus function (right Laplace transform)

4.44 $${\tilde{\psi }}_{\pi t}(\eta ,0)=\int_{0}^{\infty }{\text{e}}^{j\eta u}\psi_{t}(-u,0)\;\text{d}u.$$

## 5. Properties and validation of the functional equations

### (a) Explicit form for regions 1 and 2 and validation

Using the concept of non-standard Laplace transforms (see §1.4 in [5]), the validity of the functional equations (4.16) and (4.17), (4.24) and (4.25), (4.34) and (4.35), (4.42) and (4.43) obtained in the absence of sources is extended to the total fields in the presence of plane-wave sources or in general of sources located at infinity.

In order to validate the functional equations obtained in this paper, (4.16) and (4.17), (4.24) and (4.25), (4.34) and (4.35), (4.42) and (4.43), we demonstrate that they are equivalent to those proposed in ch. 3 of [5] for electromagnetic applications with the angular regions filled by an isotropic medium with permittivity ε and permeability μ. Let us consider, for simplicity, region 1 with (4.16) and (4.17), i.e.

5.1 $$\nu_{3}(\eta )\cdot {\tilde{\psi }}_{t}(\eta ,0)=\nu_{3}(\eta )\cdot {\overset{⌣}{\psi }}_{as}(-m_{a1}(\gamma ,\eta ))$$

and

5.2 $$\nu_{4}(\eta )\cdot {\tilde{\psi }}_{t}(\eta ,0)=\nu_{4}(\eta )\cdot {\overset{⌣}{\psi }}_{as}(-m_{a2}(\gamma ,\eta )).$$

These equations need to be compared with (3.3.57) and (3.3.58) of [5], which for readability are reported here using the notation of this paper

5.3 $$\begin{array}{rl} & \xi {\tilde{E}}_{z}(\eta ,0)-\frac{\tau_{o}^{2}}{\omega \varepsilon }{\tilde{H}}_{x}(\eta ,0)-\frac{\alpha_{o}\eta }{\omega \varepsilon }{\tilde{H}}_{z}(\eta ,0)\\ & \;=-n{\overset{⌣}{E}}_{z}(-m,\gamma )-\frac{\tau_{o}^{2}}{\omega \varepsilon }{\overset{⌣}{H}}_{\rho }(-m,\gamma )+\frac{\alpha_{o}m}{\omega \varepsilon }{\overset{⌣}{H}}_{z}(-m,\gamma ) \end{array}$$

and

5.4 $$\begin{array}{rl} & \xi {\tilde{H}}_{z}(\eta ,0)+\frac{\tau_{o}^{2}}{\omega \varepsilon }{\tilde{E}}_{x}(\eta ,0)+\frac{\alpha_{o}\eta }{\omega \varepsilon }{\tilde{E}}_{z}(\eta ,0)\\ & \;=-n{\overset{⌣}{H}}_{z}(-m,\gamma )+\frac{\tau_{o}^{2}}{\omega \varepsilon }{\overset{⌣}{E}}_{\rho }(-m,\gamma )-\frac{\alpha_{o}m}{\omega \varepsilon }{\overset{⌣}{E}}_{z}(-m,\gamma ), \end{array}$$

where for the isotropy of media

5.5 $$m=m(\gamma ,\eta )=m_{a1}(\gamma ,\eta )=m_{a2}(\gamma ,\eta )=-\eta \cos\gamma +\xi \sin\gamma =j\lambda_{e3}(\gamma ,\eta )=j\lambda_{e4}(\gamma ,\eta )$$

and

5.6 n=n(γ,η)=−ηsin⁡γ−ξcos⁡γ.

In (5.3) and (5.4), we have used Laplace transforms in *η* along u>0,v=0 on the LHS and in −m along u=0,v>0 on the RHS, respectively, denoted by ∼ and ⌣ symbols and reported in (4.1) and (4.15).

To explicitly represent (5.1) and (5.2), we apply on the LHS the definitions of $\psi_{t}=|E_{z}\;E_{x}\;H_{z}\;H_{x}{|}^{\prime }$ and the reciprocal vectors reported in (2.32).

On the RHS, we use the source term $\psi_{as}(v)$ (4.5) of the differential equation (4.3), which, substituting the explicit expressions of $M_{e1}$ and $M_{e2}$ reported in (3.6), yields

5.7 $$\psi_{as}(v)=\left|\begin{array}{c} E_{z}\cos(\gamma )+\frac{\alpha_{o}H_{z}\sin(\gamma )}{\omega \epsilon }\\ E_{x}\cos(\gamma )+\frac{jD_{u}H_{z}\sin(\gamma )-H_{x}\alpha_{o}\sin(\gamma )+H_{z}\eta \sin(\gamma )}{\omega \epsilon }\\ H_{z}\cos(\gamma )-\frac{\alpha_{o}E_{z}\sin(\gamma )}{\mu \omega }\\ H_{x}\cos(\gamma )+\frac{-jD_{u}E_{z}\sin(\gamma )+\alpha_{o}E_{x}\sin(\gamma )-E_{z}\eta \sin(\gamma )}{\mu \omega } \end{array}\right|,$$

where $D_{u}=\partial /\partial u$ and the field quantities are defined for $u=0_{+}$ and depend on v>0.

We observe that, while ${\tilde{\psi }}_{t}(\eta ,0)$ is continuous at φ=0 by definition (2.5), we need to apply mathematical manipulations to demonstrate the continuity of $\psi_{as}(v)$ (5.7) at face a for an arbitrary aperture angle *γ*. In fact, $\psi_{as}(v)$ shows possible discontinuous terms at face a ($u=0_{+},v>0$) owing to the presence of $D_{u}H_{z}$ and $D_{u}E_{z}$.

For this purpose, we resort to Maxwell’s equations

5.8 $$D_{u}H_{z}=j\frac{-kE_{y}-H_{x}Z_{o}\alpha_{o}}{Z_{o}},\;D_{u}E_{z}=j(kZ_{o}H_{y}-\alpha_{o}E_{x}).$$

Substituting (5.8), where *Z*_*o*_ is the impedance of the medium, for example free space, into (5.7)

5.9 $$\psi_{as}(v)=\left|\begin{array}{c} E_{z}\cos(\gamma )+\frac{\alpha_{o}H_{z}\sin(\gamma )}{\omega \epsilon }\\ E_{x}\cos(\gamma )+\frac{\left(kE_{y}+H_{z}\eta Z_{o})\sin(\gamma \right)}{k}\\ H_{z}\cos(\gamma )-\frac{\alpha_{o}E_{z}\sin(\gamma )}{\mu \omega }\\ H_{x}\cos(\gamma )+\frac{\left(H_{y}kZ_{o}-E_{z}\eta )\sin(\gamma \right)}{kZ_{o}} \end{array}\right|,$$

where the field quantities are defined for $u=0_{+}$ and depend on v>0. The next step is to rewrite $E_{x}$, $E_{y}$, $H_{x}$ and $H_{y}$ in terms of the components $\left(E_{v},H_{v}\right)$ and $(E_{n},H_{n}),$ respectively, tangential and normal to face a (outward normal with respect to region 1). We have

5.10 $$\begin{array}{rl} & E_{x}=-E_{n}\sin(\gamma )+E_{v}\cos(\gamma ),\\ & H_{x}=-H_{n}\sin(\gamma )+H_{v}\cos(\gamma ),\\ & E_{y}=E_{v}\sin(\gamma )+E_{n}\cos(\gamma )\\ \;\text{and}\; & H_{y}=H_{v}\sin(\gamma )+H_{n}\cos(\gamma ). \end{array}\}$$

Substituting (5.10) into (5.9), we have

5.11 $$\psi_{as}(v)=\left|\begin{array}{c} E_{z}\cos(\gamma )+\frac{\alpha_{o}H_{z}\sin(\gamma )}{\omega \epsilon }\\ E_{v}+\frac{H_{z}\eta Z_{o}\sin(\gamma )}{k}\\ H_{z}\cos(\gamma )-\frac{\alpha_{o}E_{z}\sin(\gamma )}{\mu \omega }\\ H_{v}+\frac{-E_{z}\eta \sin(\gamma )}{kZ_{o}} \end{array}\right|.$$

Note that the discontinuous components of fields (i.e. the normal components of electromagnetic field *E*,*H*) are cancelled by substitution in (5.11), thus $\psi_{as}(v)$ is continuous at face a. The absence of the discontinuous components $E_{n},H_{n}$ in (5.11) is justified by the equivalence theorem of electromagnetism, i.e. the field in region 1 can be computed and depends only on the field components continuous at the boundaries: for face a the tangential components of the electromagnetic field are $E_{v},H_{v},E_{z},H_{z}$ in u,v,z.

Now, substituting the Laplace transforms ${\tilde{\psi }}_{t}(\eta ,0)$ (4.1) of $\psi_{t}(u,0)$ and ${\overset{⌣}{\psi }}_{as}(-m)$ (4.15) of $\psi_{as}(v)$ (5.11) with (5.5) into (5.1) and (5.2), and using (2.32), yields the two functional equations

5.12 $$\begin{array}{rl} & -\alpha_{o}\eta {\tilde{E}}_{x}+(\eta^{2}-k^{2}){\tilde{E}}_{z}+k\xi Z_{o}{\tilde{H}}_{x}\\ & \;=-\alpha_{o}\eta {\overset{⌣}{E}}_{v}-[\eta \xi \sin(\gamma )+\cos(\gamma )(k^{2}-\eta^{2})]{\overset{⌣}{E}}_{z}+k\xi Z_{o}{\overset{⌣}{H}}_{v}-\sin(\gamma )\alpha_{o}kZ_{o}{\overset{⌣}{H}}_{z} \end{array}$$

and

5.13 $${\tau_{o}}^{2}{\tilde{E}}_{x}+\alpha_{o}\eta {\tilde{E}}_{z}+k\xi Z_{o}{\tilde{H}}_{z}={\tau_{o}}^{2}{\overset{⌣}{E}}_{v}+\alpha_{o}[\cos(\gamma )\eta -\sin(\gamma )\xi ]{\overset{⌣}{E}}_{z}+kZ_{o}[\sin(\gamma )\eta +\cos(\gamma )\xi ]{\overset{⌣}{H}}_{z},$$

which we have normalized by the multiplying factor $2kZ_{o}\xi$. In (5.12) and (5.13), the field quantities on the LHS are Laplace transforms in *η* along u>0,v=0 (symbol ∼), while the field quantities on the RHS are Laplace transforms in −m along v>0,u=0 (symbol ⌣). As a consequence, the field components on the LHS are plus functions in *η*, while those on the RHS are minus functions in *m*. We also observe that *v* components of the field in oblique Cartesian coordinates are equivalent to ρ components in cylindrical coordinates.

Equations (5.12) and (5.13) are explicit expressions of functional equations of region 1 filled by an isotropic medium.

We note that (5.3) and (5.4) and (5.12) and (5.13) are obtained using completely different methods and therefore equivalence is not immediate in the general case $\alpha_{o}\neq 0$. However, each of (5.12) and (5.13) is a linear combination of (5.3) and (5.4) and vice versa.

For simplicity, we explicitly report the equivalence between (5.3) and a linear combination of (5.12) and (5.13). First, we demonstrate the equivalence of the left member of (5.3) to the left member of a linear combination between (5.12) and (5.13), imposing

5.14 $$2kZ_{o}\xi (C_{1}\nu_{3}(\eta )\cdot {\tilde{\psi }}_{t}(\eta ,0)+C_{2}\nu_{4}(\eta )\cdot {\tilde{\psi }}_{t}(\eta ,0))=\xi {\tilde{E}}_{z}(\eta ,0)-\frac{\tau_{o}^{2}}{\omega \varepsilon }{\tilde{H}}_{x}(\eta ,0)-\frac{\alpha_{o}\eta }{\omega \varepsilon }{\tilde{H}}_{z}(\eta ,0).$$

To evaluate the linear combination constants $C_{1}$ and $C_{2}$ in (5.14), first we impose that the coefficients of ${\tilde{H}}_{x}$ in both the members of (5.14) are the same. This yields

5.15 $$C_{1}=-\frac{\tau_{o}^{2}}{k^{2}\xi }.$$

Second, we need to eliminate the component ${\tilde{E}}_{x}$ from the first member of (5.14) since no ${\tilde{E}}_{x}$ component is present at the second member, therefore

5.16 $$C_{2}=C_{1}\frac{\alpha_{o}\eta }{\tau_{o}^{2}}.$$

With the above values of $C_{1}$ and $C_{2}$ the identity (5.14) holds.

Finally, we simply prove by substitution that the constants (5.15) and (5.16) enforce the same equality on the right-hand members of the two formulations, i.e.

5.17 $$\begin{array}{rl} & 2kZ_{o}\xi (C_{1}\nu_{3}(\eta )\cdot {\overset{⌣}{\psi }}_{as}(-m)+C_{2}\nu_{4}(\eta )\cdot {\overset{⌣}{\psi }}_{as}(-m))\\ & \;=-n{\overset{⌣}{E}}_{z}(-m,\gamma )-\frac{\tau_{o}^{2}}{\omega \varepsilon }{\overset{⌣}{H}}_{\rho }(-m,\gamma )+\frac{\alpha_{o}m}{\omega \varepsilon }{\overset{⌣}{H}}_{z}(-m,\gamma ). \end{array}$$

Owing to the structure of (5.4), which is similar to that of (5.3), it is possible to demonstrate the equivalence of (5.4) to a linear combination of (5.15) and (5.16) with the same procedure, which we omit here.

Analogously to region 1, we can derive the explicit form of functional equations (4.24) and (4.25) for region 2 filled by an isotropic medium with permittivity ε and permeability μ

5.18 $$\nu_{1}(\eta )\cdot {\tilde{\psi }}_{t}(\eta ,0)=-\nu_{1}(\eta )\cdot {\overset{⌣}{\psi }}_{bs}(-m_{b1}(\gamma ,\eta ))$$

and

5.19 $$\nu_{2}(\eta )\cdot {\tilde{\psi }}_{t}(\eta ,0)=-\nu_{2}(\eta )\cdot {\overset{⌣}{\psi }}_{bs}(-m_{b2}(\gamma ,\eta )).$$

Regions 1 and 2 share the same procedure to obtain the explicit form of the functional equations. In particular, we note the following analogies and differences: (i) the source term assumes the same form $\psi_{bs}(v)=\psi_{as}(v)$ (5.11) with the exception of the dependence on the constitutive parameters ε,μ and (ii) while applying Maxwell’s equations (5.8) to represent the field components in terms of face a(b) tangential $\left(E_{v},H_{v}\right)$ and normal $\left(E_{n},H_{n}\right)$ components we need to consider the outward normal of region 1(2).

Focusing our attention on region 2 and substituting the Laplace transforms ${\tilde{\psi }}_{t}(\eta ,0)$ (4.1) of $\psi_{t}(u,0)$ and ${\overset{⌣}{\psi }}_{bs}(-m_{b})$ (4.23) of $\psi_{bs}(v)$ with

5.20 $$m_{b}=m_{b}(\gamma ,\eta )=m_{b1}(\gamma ,\eta )=m_{b2}(\gamma ,\eta )=\eta \cos\gamma +\xi \sin\gamma =j\lambda_{e1}(\gamma ,\eta )=j\lambda_{e2}(\gamma ,\eta )$$

into (5.18) and (5.19), and using (2.32), yields the two functional equations

5.21 $$\begin{array}{rl} & +\alpha_{o}\eta {\tilde{E}}_{x}-(\eta^{2}-k^{2}){\tilde{E}}_{z}+k\xi Z_{o}{\tilde{H}}_{x}\\ & \;=-\alpha_{o}\eta {\overset{⌣}{E}}_{v}-[-\eta \xi \sin(\gamma )+\cos(\gamma )(k^{2}-\eta^{2})]{\overset{⌣}{E}}_{z}-k\xi Z_{o}{\overset{⌣}{H}}_{v}-\sin(\gamma )\alpha_{o}kZ_{o}{\overset{⌣}{H}}_{z} \end{array}$$

and

5.22 $$-{\tau_{o}}^{2}{\tilde{E}}_{x}-\alpha_{o}\eta {\tilde{E}}_{z}+k\xi Z_{o}{\tilde{H}}_{z}={\tau_{o}}^{2}{\overset{⌣}{E}}_{v}+\alpha_{o}[\cos(\gamma )\eta +\sin(\gamma )\xi ]{\overset{⌣}{E}}_{z}+kZ_{o}[\sin(\gamma )\eta -\cos(\gamma )\xi ]{\overset{⌣}{H}}_{z},$$

which we have normalized with the multiplying factor $2kZ_{o}\xi$. Equations (5.21) and (5.22) show a change in sign with respect to (5.12) and (5.13) of region 1. In (5.21) and (5.22), the field quantities on the LHS are Laplace transforms in *η* along u>0,v=0 (symbol ∼), while the field quantities on the RHS are Laplace transforms in $-m_{b}$ along v<0,u=0 (symbol ⌣). As a consequence, the field components on the LHS are plus functions in *η*, while those on the RHS are minus functions in $m_{b}$. We also observe that *v* components of a field in oblique Cartesian coordinates are equivalent to ρ components with the opposite sign in cylindrical coordinates (the sign is due to the face b orientation; see figure 1). The equivalence of (5.21) and (5.22) to (3.3.59) and (3.3.60) of [5] can be accomplished as already done for (5.3), which is a linear combination of (5.12) and (5.13). In this case, we need to pay attention that *γ* in (5.21) and (5.22) must be substituted by $\pi -\gamma_{b}$ for the equivalence with (3.3.59) and (3.3.60) of [5], since figure 1 of this paper describes a region 2 that is different from the one in figure 3.3.2 in [5]. Moreover, explicit expressions of functional equations for more complex media can be derived starting from the definitions of $M_{m}$ matrices in (2.22): in appendix A, we report the matrices for the anisotropic case.

### (b) A classical example of generalized Wiener–Hopf equations for the validation of functional equations: the Malyuzhinets problem

In this subsection, to further convince readers about the validity and the correctness of the proposed procedure based on the matrix first-order differential formulation (§4), we derive the GWHEs for a classical scalar problem: the Malyuzhinets problem.

The general derivations of functional equations of the angular regions do not depend on the materials, the sources located outside the considered angular region or the boundary conditions.

By imposing on them the constitutive parameters of the media and the boundary conditions on the faces, we get GWHEs that in general are coupled to the electromagnetic equations present in the regions outside the considered angular region.

We affirm that, in particular, the functional equations are useful for deriving GWHEs for wedge problems with impenetrable boundaries as well as for those with penetrable ones; see for instance applications in [6,7]. Moreover, the functional equations of angular regions can be used to describe more complex scattering problems where angular regions are coupled with stratified planar regions; see for instance [8,9].

If we are interested in decoupling the evaluation of the electromagnetic field in a region from the equations that hold outside, we can resort to impenetrable approximate boundary conditions.

For instance, we can assume Leontovich boundary conditions that impose impedance surfaces on the faces of the angular region [40]. In this context, several studies have been developed based on higher order approximate boundary conditions that involve derivatives of the components of the field on the faces. In particular, these enhanced versions of boundary conditions have been examined in right-angled structures [18–20], yielding RH problems with exact solutions.

In this section, we report, as a simple demonstration of the method, the classical impenetrable wedge-scattering problem known as the Malyuzhinets problem [41], which is extensively studied in the literature using different methods. We start from the functional equations and we derive the GWHEs of the problem.

**Figure 2.**
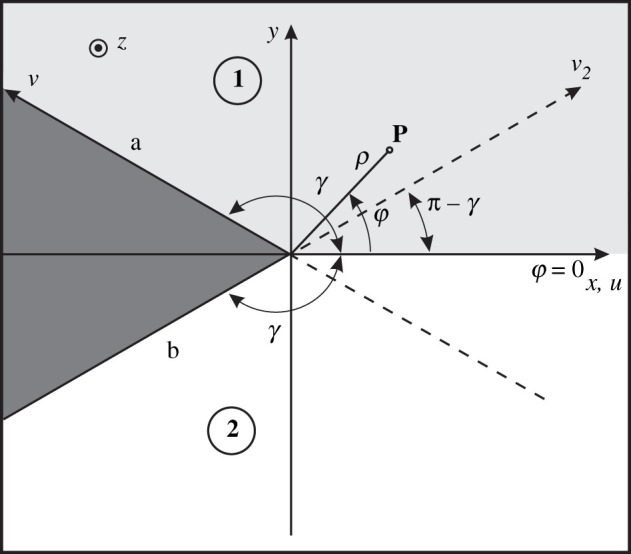
Impenetrable wedge problem with surrounding space made by a homogeneous isotropic medium divided into angular regions 1 and 2. Cartesian coordinates (x,y,z) and cylindrical coordinates (ρ,φ,z) are reported. For each angular region, a local oblique Cartesian coordinate system is defined: for region 1 u,v,z with aperture angle *γ*, for region 2 $u,v_{2},z$ with aperture angle π−γ. With respect to figure 1, regions 3 and 4 are not physically considered. Boundary conditions are imposed at faces a and b.

With reference to figure 2, the Malyuzhinets problem consists of an impenetrable wedge structure immersed in an isotropic medium and illuminated by a plane wave at normal incidence ($\alpha_{o}=0$), where the following scalar boundary conditions are imposed in cylindrical coordinates:

5.23 $$\left[\begin{array}{c} E_{z}(\rho ,\gamma )\\ E_{\rho }(\rho ,\gamma ) \end{array}\right]=Z_{a}\left[\begin{array}{c} H_{\rho }(\rho ,\gamma )\\ -H_{z}(\rho ,\gamma ) \end{array}\right],\;\left[\begin{array}{c} E_{z}(\rho ,-\gamma )\\ E_{\rho }(\rho ,-\gamma ) \end{array}\right]=-Z_{b}\left[\begin{array}{c} H_{\rho }(\rho ,-\gamma )\\ -H_{z}(\rho ,-\gamma ) \end{array}\right].$$

In figure 2, with respect to figure 1, we identify two symmetrical homogeneous isotropic regions, respectively, with aperture angle *γ* and π−γ, while regions 3 and 4 are not physically considered.

The functional equations of region 1 are reported in (5.12) and (5.13), before the application of the boundary conditions of the problem. For region 2, we note the difference in the aperture angle in figure 1 with respect to the aperture angle in figure 2. For this reason, to derive the functional equations of region 2 in figure 2, we need to replace *γ* with π−γ in (5.21) and (5.22).

At normal incidence ($\alpha_{o}=0$), the functional equations of region 1 take the following form:

5.24 $$\xi {\tilde{E}}_{z}+kZ_{o}{\tilde{H}}_{\rho }=-[\eta \sin(\gamma )+\cos(\gamma )\xi ]{\overset{⌣}{E}}_{z}+kZ_{o}{\overset{⌣}{H}}_{\rho }$$

and

5.25 $$k{\tilde{E}}_{\rho }+\xi Z_{o}{\tilde{H}}_{z}=k{\overset{⌣}{E}}_{\rho }+Z_{o}[\sin(\gamma )\eta +\cos(\gamma )\xi ]{\overset{⌣}{H}}_{z},$$

with direction vectors $\hat{v}=\hat{\rho }$ for φ=γ (face a) and $\hat{x}=\hat{\rho }$ for φ=0. The field quantities on the LHS of (5.24) and (5.25) depend on *η* and are evaluated for φ=0, i.e. $\tilde{F}=\tilde{F}(\eta ,\varphi =0)$, while the field quantities on the RHS depend on −m (5.5) and are evaluated for φ=γ, i.e. $\overset{⌣}{F}=\overset{⌣}{F}(-m,\varphi =+\gamma )$.

The functional equations in region 2 take the following form:

5.26 $$\xi {\tilde{E}}_{z}+kZ_{o}{\tilde{H}}_{\rho }=[\eta \sin(\gamma )+\cos(\gamma )\xi ]{\overset{⌣}{E}}_{z}+kZ_{o}{\overset{⌣}{H}}_{\rho }$$

and

5.27 $$-k{\tilde{E}}_{\rho }+\xi Z_{o}{\tilde{H}}_{z}=-k{\overset{⌣}{E}}_{\rho }+Z_{o}[\sin(\gamma )\eta +\cos(\gamma )\xi ]{\overset{⌣}{H}}_{z},$$

with direction vectors ${\hat{v}}_{2}=-\hat{\rho }$ for φ=−γ (face b) and $\hat{x}=\hat{\rho }$ for φ=0 (see figure 2). Note also that for region 2 in figure 2 we have, from (5.20) and (5.5),

5.28 $$m_{b}(\pi -\gamma ,\eta )=-\eta \cos\gamma +\xi \sin\gamma =m.$$

In this case, while the field quantities on the LHS of (5.26) and (5.27) are the same as those on the LHS of (5.24) and (5.25), i.e. $\tilde{F}=\tilde{F}(\eta ,\varphi =0)$, the field quantities on the RHS of (5.26) and (5.27) depend on −m and are evaluated for φ=−γ, i.e. $\overset{⌣}{F}=\overset{⌣}{F}(-m,\varphi =-\gamma )$.

For simplicity, focusing our attention on $E_{z}$ polarization, we use only (5.24) and (5.26). By imposing the boundary conditions (5.23) and eliminating ${\overset{⌣}{E}}_{z}$, we obtain the following system of equations after some mathematical manipulation:

5.29 $$-\xi {\tilde{E}}_{z}(\eta ,0)+kZ_{o}{\tilde{H}}_{\rho }(\eta ,0)=(kZ_{o}+nZ_{a}){\overset{⌣}{H}}_{\rho }(-m,+\gamma )$$

and

5.30 $$\xi {\tilde{E}}_{z}(\eta ,0)+kZ_{o}{\tilde{H}}_{\rho }(\eta ,0)=(kZ_{o}+nZ_{b}){\overset{⌣}{H}}_{\rho }(-m,-\gamma ),$$

with n=−ηsin⁡(γ)−cos⁡(γ)ξ. Finally, (5.29) and (5.30) can be reduced in the normal form to

5.31 $$G(\eta )F_{+}(\eta )=F_{-}(m)$$

with

5.32 $$\begin{array}{rl} & G(\eta )=\left|\begin{array}{cc} -\frac{\xi }{Z_{a}(n_{a}+n)} & \frac{kZ_{o}}{Z_{a}(n_{a}+n)}\\ \frac{\xi }{Z_{b}(n_{b}+n)} & \frac{kZ_{o}}{Z_{b}(n_{b}+n)} \end{array}\right|,\;F_{+}(\eta )=\left|\begin{array}{c} {\tilde{E}}_{z}(\eta ,0)\\ {\tilde{H}}_{\rho }(\eta ,0) \end{array}\right|\\ \;\text{and}\; & F_{-}(m)=\left|\begin{array}{c} {\overset{⌣}{H}}_{\rho }(-m,+\gamma )\\ {\overset{⌣}{H}}_{\rho }(-m,-\gamma ) \end{array}\right| \end{array}\}$$

and where $n_{a,b}=kZ_{o}/Z_{a,b}$. Note that (5.31) is a matrix GWHE with kernel G(η), plus functions $F_{+}(\eta )$ in *η* and minus functions $F_{-}(m)$ in *m*. Solutions to the GWHEs of the Malyuzhinets problem can be found in [2–6,10] using analytical and/or semi-analytical procedures after their reduction to CWHEs in a new complex plane $\bar{\eta }$ using the special mapping [5]

5.33 $$\eta (\bar{\eta })=-k\cos\left(-\frac{\gamma }{\pi }\arccos\left(-\frac{\bar{\eta }}{k}\right)\right).$$

### (c) Remarks on the functional equations to obtain generalized Wiener–Hopf equations

In general, the functional equations (4.16) and (4.17), (4.24) and (4.25), (4.34) and (4.35), (4.42) and (4.43), respectively, for regions 1–4 (figure 1) are the starting point for deriving the GWHEs of arbitrary angular regions (aperture angle, material) in complex scattering problems. In order to obtain the GWHEs for a practical problem, we need to define the media and to enforce the boundary conditions at the interfaces of the regions. For instance, see electromagnetic scattering problems by anisotropic impedance wedges in [4,6], §5.2 in [10] and more complex problems in [7–10].

With reference to figure 1, we observe that the *axial spectra* ${\tilde{\psi }}_{t}(\eta ,0)$ and ${\tilde{\psi }}_{\pi t}(\eta ,0)$ at the interfaces, respectively, between regions 1 and 2 and between regions 3 and 4 are defined in terms of only continuous components of the fields satisfying the boundary conditions in electromagnetic problems. Meanwhile, the *face spectra* ${\overset{⌣}{\psi }}_{s}(\chi )$ on the interface between regions 1 and 4 (2 and 3) could present discontinuous components and/or derivatives of the fields; see faces a and d (faces b and c) in figure 1. To check the continuity of the face spectra, we have re-written the component of ${\overset{⌣}{\psi }}_{s}(\chi )$ in terms of continuous components of the field in the case of isotropic media. In a practical case, according to our experience, we note that appropriate relations are always available in arbitrary linear media.

Once the GWHEs have been obtained from the functional equations of an angular region problem, an important aspect is their reduction to CWHEs by using a suitable mapping, such as the one reported in (5.33).

The introduction of the complex angular plane *w*

5.34 η=−kcos⁡w

helps the analysis of asymptotic solutions to practical problems by allowing analytical extension of the approximate solutions [5–10]. In fact, the application of (5.34) to GWHEs allows us to obtain difference equations that are useful for recursive applications. We further note that the difference equations relate GWHEs to the SM method for a valuable synergy between the two methods.

This article reports explicit expressions of functional equations for isotropic media. However, the procedure is general and applicable to more complex media, starting from the definitions of $M_{m}$ matrices in (2.22); in appendix A, we report the matrices for the anisotropic media.

## 6. Conclusion

In this work, we have introduced a general method for the deduction of spectral functional equations in angular regions filled by arbitrary linear homogeneous media. These equations are obtained by solving vector differential equations of first order using the dyadic Green’s function and then by projecting the solution on reciprocal eigenvectors of an algebraic matrix related to the medium filling the angular region. The fundamental starting point to derive equations in arbitrary linear media is the derivation of matrices $M_{o},M_{1},M_{2}$. From a practical point of view, we have reported these matrices for anisotropic media in appendix A, while the main text contains those for isotropic media. The derivation of explicit equations requires the implementation of the procedure reported in the paper, illustrated explicitly for isotropic media. The application of the boundary conditions to the functional equations yields GWHEs for practical problems. In this paper, the method is applied to electromagnetic applications and the functional equations are explicitly derived and verified in the case of isotropic media with respect to the current literature.

The efficacy of the GWHE formulation has been demonstrated in several practical electromagnetic engineering works by the authors; see the references. We assert that the proposed method to obtain spectral functional equations in arbitrary angular regions for the wave motion problem is general and is applicable to different physics.

## Figures and Tables

**Table 1.** Main abbreviations, notations and symbols introduced in the paper.

notation	description
WH	Wiener–Hopf
GWHEs	generalized Wiener–Hopf equations
CWHEs	classical Wiener–Hopf equations
SM	Sommerfeld–Malyuzhinets (method)
PEC	perfect electrical conductor
(x,y,z)	Cartesian coordinates
(ρ,φ,z)	cylindrical coordinates
(u,v,z)	oblique Cartesian coordinates
E, H, D, B	electric field, magnetic field, dielectric induction, magnetic induction
k	propagation constant
$Z_{o}$	free space impedance
ε,μ and ξ,ζ	tensor constitutive parameters (electric permittivity, magnetic permeability and magnetoelectric tensors)
${\text{e}}^{j\omega t}$	time dependence of a harmonic field
$\Gamma_{\nabla }$	matrix differential operator in abstract notation
ψ, θ	vector fields in abstract notation
*W*	matrix constitutive parameters of media
$\psi_{t}$	transverse field for a stratification along the *y*-direction
$\psi_{y}$	longitudinal field for a stratification along the *y*-direction
M(∂/∂z,∂/∂x)	transversal operator for Maxwell’s equations
$D_{x}=\partial /\partial x$	alternative partial derivative notation
$\alpha_{o}$	due to invariance along the *z*-direction, without loss of generality, we suppose that a field dependence specified by the factor $\;{\text{e}}^{-j\alpha_{o}\;z}$
*η*	Fourier or Laplace spectral variable according to the position in the text
$\Psi_{t}(\eta )$	Fourier transform along the x=u direction (y,z or v,z dependence is omitted)
M(η)	matrix operator in the Fourier/Laplace domain in an indefinite rectangular region
$\lambda_{i},\;u_{i}$	eigenvalues and eigenvector of M(η)
$\nu_{i}$	reciprocal vectors of $u_{i}$
$\xi_{i}$	different notation of $\lambda_{i}$ for propagation’s properties, multivalued function
*γ*	aperture angle of angular regions (figure 1)
$M_{e}(\gamma ,\eta )$	matrix operator in the Fourier/Laplace domain in an indefinite angular region
$\lambda_{ei}$	eigenvalues of $M_{e}(\gamma ,\eta )$
${\tilde{\psi }}_{t}(\eta ,v)$	Laplace transform along x≡u of $\psi_{t}(u,v)$ (omitting *z* dependence)
$\psi_{s}(v)$	field components on the face of an angular region in the Laplace domain
$\psi_{as}(v)$	specialized expression of $\psi_{s}(v)$ on face a
$G(v,v^{\prime })$	dyadic Green’s function in the Laplace domain for an angular region
${\overset{⌣}{\psi }}_{as}(\chi )$	Laplace transform in *v* along face a (v=ρ)
$m_{a1},m_{a2}$	spectral variables for the evaluation of ${\overset{⌣}{\psi }}_{as}(\chi )$ in functional equations

## Data accessibility

This article has no additional data.

## Appendix A

In this appendix, we report the explicit definitions of the fundamental matrices $M_{o}$, $M_{1}$, $M_{2}$ (2.22) that are useful for developing applications of the method in electromagnetic anisotropic media, i.e. ξ=0, ζ=0 in (2.3) and (2.4) (we avoid reporting the matrices for the bi-anisotropic case because of their length). In particular, to develop the procedure, it is sufficient to replace (2.23) of the isotropic case with

A 1 $$\begin{array}{rl} M_{o} & =\left|\begin{array}{cccc} -\frac{j\alpha_{o}\varepsilon_{yz}}{\varepsilon_{yy}} & -j\alpha_{o}\left(\frac{\varepsilon_{yx}}{\varepsilon_{yy}}-\frac{\mu_{xy}}{\mu_{yy}}\right) & \frac{j\omega (\mu_{xz}\mu_{yy}-\mu_{xy}\mu_{yz})}{\mu_{yy}} & j\omega \left(\mu_{xx}-\frac{\mu_{xy}\mu_{yx}}{\mu_{yy}}\right)-\frac{j{\alpha_{o}}^{2}}{\varepsilon_{yy}\omega }\\ 0 & -\frac{j\alpha_{o}\mu_{zy}}{\mu_{yy}} & \frac{j\omega (\mu_{yz}\mu_{zy}-\mu_{yy}\mu_{zz})}{\mu_{yy}} & -j\omega \left(\mu_{zx}-\frac{\mu_{yx}\mu_{zy}}{\mu_{yy}}\right)\\ -j\omega \left(\varepsilon_{xz}-\frac{\varepsilon_{xy}\varepsilon_{yz}}{\varepsilon_{yy}}\right) & \frac{j{\alpha_{o}}^{2}}{\mu_{yy}\omega }+\frac{j\omega (\varepsilon_{xy}\varepsilon_{yx}-\varepsilon_{xx}\varepsilon_{yy})}{\varepsilon_{yy}} & -\frac{j\alpha_{o}\mu_{yz}}{\mu_{yy}} & j\alpha_{o}\left(\frac{\varepsilon_{xy}}{\varepsilon_{yy}}-\frac{\mu_{yx}}{\mu_{yy}}\right)\\ \frac{j\omega (\varepsilon_{yy}\varepsilon_{zz}-\varepsilon_{yz}\varepsilon_{zy})}{\varepsilon_{yy}} & \frac{j\omega (\varepsilon_{yy}\varepsilon_{zx}-\varepsilon_{yx}\varepsilon_{zy})}{\varepsilon_{yy}} & 0 & -\frac{j\alpha_{o}\varepsilon_{zy}}{\varepsilon_{yy}} \end{array}\right|, \end{array}$$

A 2 $$\begin{array}{rl} M_{1} & =\left|\begin{array}{cccc} -\frac{j\mu_{xy}}{\mu_{yy}} & 0 & \frac{j\alpha_{o}}{\varepsilon_{yy}\omega } & 0\\ \frac{j(\varepsilon_{yy}\mu_{zy}-\varepsilon_{yz}\mu_{yy})}{\varepsilon_{yy}\mu_{yy}} & -\frac{j\varepsilon_{yx}}{\varepsilon_{yy}} & 0 & -\frac{j\alpha_{o}}{\varepsilon_{yy}\omega }\\ -\frac{j\alpha_{o}}{\mu_{yy}\omega } & 0 & -\frac{j\varepsilon_{xy}}{\varepsilon_{yy}} & 0\\ 0 & \frac{j\alpha_{o}}{\mu_{yy}\omega } & \frac{j(\varepsilon_{zy}\mu_{yy}-\varepsilon_{yy}\mu_{yz})}{\varepsilon_{yy}\mu_{yy}} & -\frac{j\mu_{yx}}{\mu_{yy}} \end{array}\right| \end{array}$$

A 3 $$\begin{array}{rl} \;\text{and}\;M_{2} & =\left|\begin{array}{cccc} -\frac{j\mu_{xy}}{\mu_{yy}} & 0 & \frac{j\alpha_{o}}{\varepsilon_{yy}\omega } & 0\\ \frac{j(\varepsilon_{yy}\mu_{zy}-\varepsilon_{yz}\mu_{yy})}{\varepsilon_{yy}\mu_{yy}} & -\frac{j\varepsilon_{yx}}{\varepsilon_{yy}} & 0 & -\frac{j\alpha_{o}}{\varepsilon_{yy}\omega }\\ -\frac{j\alpha_{o}}{\mu_{yy}\omega } & 0 & -\frac{j\varepsilon_{xy}}{\varepsilon_{yy}} & 0\\ 0 & \frac{j\alpha_{o}}{\mu_{yy}\omega } & \frac{j(\varepsilon_{zy}\mu_{yy}-\varepsilon_{yy}\mu_{yz})}{\varepsilon_{yy}\mu_{yy}} & -\frac{j\mu_{yx}}{\mu_{yy}} \end{array}\right|. \end{array}$$

As a practical propagation example, by restricting the case to $\alpha_{o}=0$ and diagonal ε, μ we compute easily the eigenvalues (2.27) of M(η) (2.25), yielding

A 4 $$\xi_{1}=\xi_{3}=\frac{\sqrt{\mu_{xx}}}{\sqrt{\mu_{yy}}}\sqrt{\omega^{2}\varepsilon_{zz}\mu_{yy}-\eta^{2}},\;\xi_{2}=\xi_{4}=\frac{\sqrt{\varepsilon_{xx}}}{\sqrt{\varepsilon_{yy}}}\sqrt{\omega^{2}\varepsilon_{yy}\mu_{zz}-\eta^{2}},$$

which constitutes two propagation modalities: the ordinary and extraordinary waves.

## Appendix B

In this appendix, we report the justification and the properties of the dyadic Green’s function (4.11) to get the particular solution (4.9) of (4.3). The dyadic Green’s function is the solution to the dyadic equation

B 1 $$\frac{\text{d}}{\text{d}v}G(v,v^{\prime })+M_{e}(\gamma ,\eta )G(v,v^{\prime })=\delta (v-v^{\prime })1_{t},$$

where $1_{t}$ is the unitary dyadic (2.31). According to [39], we select as solutions to the homogeneous equations to build the dyadic Green’s function progressive and regressive waves in an indefinite region. Moreover, the dyadic Green’s functions need to model the behaviour at $v=v^{\prime }$ of (B1) to allow the particular solution (4.9) to be the solution of (4.3). Using dyadic notation, for $v>v^{\prime },$ we have the set of progressive waves (i=1,2), while for $v<v^{\prime }$ we have the set of regressive waves (i=3,4), i.e.

B 2 $$G_{i}(v,v^{\prime })=u_{i}A_{i}(v^{\prime })\;{\text{e}}^{-\lambda_{ei}(\gamma ,\eta )v},\;i=1\ldots 4,$$

where $\lambda_{ei}(\gamma ,\eta )$ and $u_{i}$ are the eigenvalues and the eigenvectors of the matrix of dimension 4 and $M_{e}(\gamma ,\eta )$ and $A_{i}(v^{\prime })$ are arbitrary vector coefficients.

The most general solution of (B1) is expressed by the dyadics

B 3 $$G(v,v^{\prime })=\left\{ \begin{array}{l} \vec{G}(v,v^{\prime })=u_{1}A_{1}(v^{\prime })\;{\text{e}}^{-\lambda_{e1}(\gamma ,\eta )v}+u_{2}A_{2}(v^{\prime })\;{\text{e}}^{-\lambda_{e2}(\gamma ,\eta )v},\;v>v^{\prime },\\ \overset{\leftarrow }{G}(v,v^{\prime })=u_{3}A_{3}(v^{\prime })\;{\text{e}}^{-\lambda_{e3}(\gamma ,\eta )v}+u_{4}A_{4}(v^{\prime })\;{\text{e}}^{-\lambda_{e4}(\gamma ,\eta )v},\;v<v^{\prime }. \end{array}\right.$$

In order to find the vectors $A_{i}(v^{\prime })$, $G(v,v^{\prime })$ must satisfy (B1) also at $v=v^{\prime }$ by imposing the fundamental jump condition

B 4 $$\vec{G}(v{\;}_{+}^{\prime },v^{\prime })-\overset{\leftarrow }{G}(v{\;}_{-}^{\prime },v^{\prime })=1_{t}.$$

This yields

B 5 $$\begin{array}{rl} & u_{1}A_{1}(v^{\prime })\;{\text{e}}^{-\lambda_{e1}(\gamma ,\eta )v^{\prime }}+u_{2}A_{2}(v^{\prime })\;{\text{e}}^{-\lambda_{e2}(\gamma ,\eta )v^{\prime }}\\ & \;-(u_{3}A_{3}(v^{\prime })\;{\text{e}}^{-\lambda_{e3}(\gamma ,\eta )v^{\prime }}+u_{4}A_{4}(v^{\prime })\;{\text{e}}^{-\lambda_{e4}(\gamma ,\eta )v^{\prime }})=1_{t}. \end{array}$$

Pre-multiplying (B5) by the reciprocal eigenvectors $\nu_{i}$ (2.30) and (2.31), we get

B 6 $$\begin{array}{rl} & A_{i}(v^{\prime })=\nu_{i}\;{\text{e}}^{\lambda_{ei}(\gamma ,\eta )v^{\prime }}\;(i=1,2)\\ \;\text{and}\; & A_{i}(v^{\prime })=-\nu_{i}\;{\text{e}}^{\lambda_{ei}(\gamma ,\eta )v^{\prime }}\;(i=3,4). \end{array}\}$$

Substituting (B6) into (B3), we get the dyadic Green’s function (4.11).

## Glossary

In table 1, we report a glossary of main abbreviations, notations and symbols introduced in the paper and that are useful for its readability.
